# Physiological characteristics and root exudate responses of *Leymus chinensis* to saline-alkali stress during the regreening stage

**DOI:** 10.3389/fpls.2026.1844760

**Published:** 2026-07-07

**Authors:** Chaorong Liu, Yongcheng Chen, Ying Chen, Xudong Zhang, Tianyu Hu, Lihe Su, Rongzheng Huang, Fanfan Zhang, Hui Liu, Xuzhe Wang, Chunhui Ma

**Affiliations:** College of Animal Science and Technology, Shihezi University, Shihezi, China

**Keywords:** ion homeostasis, *Leymus chinensis*, metabolomics, root exudates, saline-alkali stress

## Abstract

**Introduction:**

Saline-alkali soil significantly inhibits the growth and development of *Leymus chinensis*, and root exudates are vital components in plant responses to abiotic stress. At present, whether *L. chinensis* accumulates specific root exudates to adapt to saline-alkali environments remains poorly understood.

**Methods:**

Therefore, in this study, the relatively saline-alkali-tolerant *L. chinensis* cultivar 'Huise' (HS) and the relatively saline-alkali-sensitive cultivar 'Dongbei' (DB) were selected as experimental materials. Three saline-alkali stress treatments were established: non-saline-alkali soil (Control, CK), moderate saline-alkali soil (MS), and severe saline-alkali soil (ES), to explore cultivar differences in physiological characteristics and root exudate metabolism under saline-alkali stress.

**Results:**

The results showed that cultivar differences in aboveground biomass were observed between HS and DB *L. chinensis* under saline-alkali stress. Intracellular Na+ accumulation and K+ decline occurred, which aggravated membrane lipid peroxidation and a 31-38% decrease in root vitality. Compared with HS, DB suffered more severe membrane lipid damage and a larger decline in root vitality. The two cultivars alleviated saline-alkali stress by simultaneously increasing the activities of peroxidase (POD), catalase (CAT), and superoxide dismutase (SOD) and accumulating proline (Pro), soluble sugars (SS), and soluble protein (SP). In total, 5014 metabolites were identified using ultra-performance liquid chromatography-tandem mass spectrometry (UPLC-MS/MS). Thirteen differentially accumulated metabolite (DAM) markers (including [8]-Shogaol, Pikuroside, L-dihydroanticapsin) were screened. Distinct cultivar differences were also observed in the number of differential root exudate metabolites under saline-alkali stress. KEGG pathway enrichment analysis revealed that the pentose phosphate pathway (PPP) and the tricarboxylic acid cycle (TCA) were significantly enriched in *L. chinensis* under saline-alkali stress. Compared with the DB cultivar, relevant biosynthetic pathways for leucine and glycine were additionally enriched in the HS cultivar.

**Discussion:**

This study identifies inter-cultivar differences in the content, composition and enriched metabolic pathways of root exudates in *L. chinensis*. These findings offer fundamental references for further exploring how saline-alkali stress correlates with physiological traits, growth performance and root exudate profiles of regreening stage *L. chinensis*.

## Graphical Abstract

**Figure:**
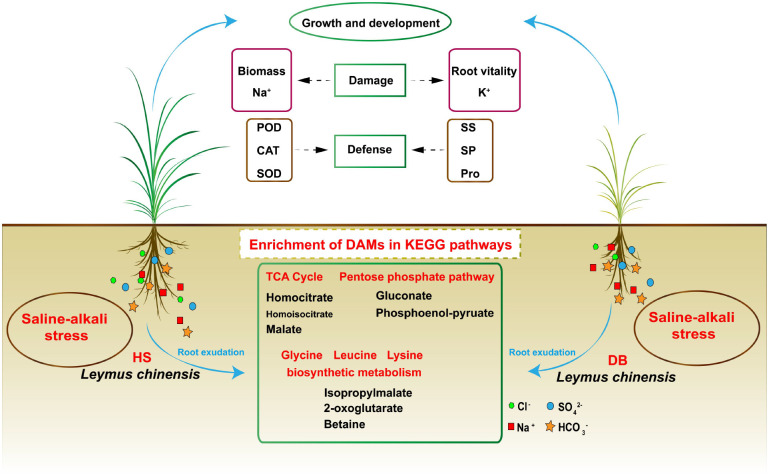

## 1 Introduction

Soil salinization has led to continuous shrinkage of global available arable land and is currently regarded as one of the most severe ecological challenges worldwide (Sun et al., 2017). The total area of saline-alkali soils in China is 3.6×10^7^ hm², with Xinjiang being a primary distribution region for such soils (De Pascale et al., 2012). The area of saline-alkali land in Xinjiang is 2.96×10^6^ hm², accounting for 42.07% of the region’s total cultivated land. According to soil salinization classification standards, slightly saline-alkali cultivated land and moderately to severely saline-alkali cultivated land account for 40% and 60%, respectively (Lyu et al., 2024).

Phytoremediation, as an environmentally sustainable soil remediation strategy, has become a key research focus in the improvement of saline-alkali soils (Ali et al., 2020; Liu et al., 2018). Therefore, promoting suitable cultivars adapted to saline-alkali soils has become a primary goal in current soil management strategies. *Leymus chinensis*, a pioneer species for the remediation and utilization of saline-alkali soils, exhibits strong stress tolerance and excellent forage characteristics (e.g., high yield and good palatability), and can survive in saline-alkali soils with a pH range of 8.5-11.5 (Wang et al., 2020). Its high saline-alkali tolerance stems from coordinated structural and physiological adaptations of roots, stems, and leaves (Chen et al., 2019; Shoji et al., 2006).

Single salt stress causes plant damage mainly via ionic and osmotic injury, while compound saline-alkali stress in natural soil environments exerts superimposed adverse effects, including ionic toxicity, osmotic water loss, oxidative damage, and high-pH alkaline stress. Such complex stress causes severe damage and pronounced growth inhibition, and induces persistent stress injuries in plants, mainly involving osmotic stress, ionic toxicity, and oxidative stress (Iqbal et al., 2015), thereby triggering a series of morphological, physiological, metabolic, and molecular adaptive changes (Yang and Guo, 2018). For example, in *Solanum lycopersicum* (Wang et al., 2011) and *Sorghum bicolor* (Sun et al., 2019), osmotic and oxidative stress induce electrolyte leakage, leading to increased relative electrical conductivity (REC) and malondialdehyde (MDA) contents, as well as aggravated membrane lipid peroxidation. Among these stresses, ionic toxicity is particularly prominent, especially in non-halophytes such as *Triticum aestivum*. Excessive Na^+^ accumulates in roots, restricting Na^+^ translocation to aboveground vegetative organs (Guo et al., 2009), which in turn decreases root vitality (Kaiwen et al., 2020) and inhibits the activity of ion transporters (Bacha et al., 2015). To cope with saline-alkali stress, plants have evolved a series of sophisticated adaptive mechanisms. (1) Morphological strategies: *S. bicolor* (Sun et al., 2019) and *L. chinensis* (Liu et al., 2022) regulate growth by modulating aboveground biomass allocation. (2) Physiological strategies: Adaptive measures such as maintaining a low Na^+^/K^+^ ratio, accumulating osmotic adjustment substances (Demidchik, 2014), and activating the antioxidant enzyme system (Liu et al., 2020) can effectively mitigate saline-alkali-induced damage. However, most previous studies on *L. chinensis* have focused on germination (Hongna et al., 2021a), seedling (Yan et al., 2022), and tillering stages (Yan et al., 2023). The regreening stage is a critical and sensitive period for forage growth, root reconstruction, and environmental acclimation, and is highly vulnerable to saline-alkali environments. Widespread mortality of *L. chinensis* is frequently found when cultivating this species on large-area saline-alkali fields during the regreening stage, yet relevant physiological and molecular basis associated with cultivar divergent stress responses remain poorly characterized. Accordingly, systematic research focusing on regreening stage *L. chinensis* responses to saline-alkali stress carries important theoretical and practical value.

Root exudates act as a key interactive linkage for plants responding to saline-alkali stress and function as the core medium connecting plant roots and the rhizosphere environment. Previous studies have demonstrated that saline-alkali stress remodels root physiological metabolism and stimulates massive secretion of organic compounds, and such exudation is hypothesized to alleviate saline-alkali phytotoxicity (Wang et al., 2024a). Root exudates possess complex chemical components, mainly consisting of carbohydrates, amino acids, organic acids, and other primary metabolites. Their contents and secretion rates are jointly affected by plant species, growth stages, and external environmental conditions (Jakoby et al., 2020). Meanwhile, root exudates can specifically recruit beneficial rhizosphere microorganisms, which is potentially associated with improved plant stress adaptability (Sasse et al., 2018). For example, high pH not only disrupts intracellular pH homeostasis in plant cells but also induces mineral ion precipitation in the rhizosphere, which restricts plant growth and photosynthetic capacity (Kaiwen et al., 2020). Under saline-alkali stress, *T. aestivum* exhibits altered rhizosphere pH accompanied by elevated carboxylic acid secretion (Wang et al., 2024b); in the presence of heavy metal stress, increased exudation of electrolytes and soluble sugars coincides with reduced stress injury (Wang et al., 2006). However, most studies on the stress tolerance of *L. chinensis* have focused on exogenous regulation strategies, such as the application of melatonin (Fan et al., 2024), salicylic acid (Hongna et al., 2021b), citric acid (Sun and Hong, 2011), and spermidine (Hongna et al., 2021a), to alleviate saline-alkali-induced damage. In contrast, limited research addresses cultivar-specific divergence in *L. chinensis*’ root exudate related stress responses, exudate profiles and corresponding metabolic pathways under saline-alkali stress. In view of the above research gaps, this study put forward the following hypotheses: (1) Under saline-alkali stress, *L. chinensis* cultivars with distinct saline-alkali tolerance exhibit significant differences in physiological responses and ion homeostasis. (2) Saline-alkali stress reshapes the root exudate metabolic profiles of *L. chinensis* during the regreening stage; tolerant and sensitive cultivars display clear divergence in the composition, content, and functional pathways enriched by root exudate-derived differentially accumulated metabolites.

This study focused on the regreening stage and compared two *L. chinensis* cultivars with contrasting saline-alkali tolerance, aiming to characterize cultivar-specific physiological and metabolic responses to saline-alkali stress from the perspective of root exudate metabolism. The core objectives were to clarify whether *L. chinensis* cultivars with divergent tolerance exhibit distinct physiological performances and metabolic profiles under saline-alkali stress, and whether their root exudate components and associated metabolic pathways show cultivar-dependent variations. To simulate the natural saline-alkali habitat of *L. chinensis* in Xinjiang, *in-situ* field soil samples were collected to ensure that soil ion compositions were consistent with actual field conditions. By integrating analyses of growth, photosynthetic and physiological traits, together with root exudate metabolomics, this study systematically investigated cultivar differences in physiological responses and root exudate variations under saline-alkali stress. This study clarifies the inter-cultivar divergence in the content, composition, and metabolic characteristics of *L. chinensis* root exudates, providing a fundamental reference for further exploring the correlations between saline-alkali environments and growth, physiological adaptation, and root exudate profiles of regreening stage *L. chinensis*.

## 2 Materials and methods

### 2.1 Plant materials and culture methods

Through a preliminary study on the differences in saline-alkali tolerance among different *L. chinensis* cultivars under saline-alkali soil conditions, it was found that there was a significant difference in saline-alkali tolerance between the ‘Huise’ cultivar (HS) and the ‘Dongbei’ cultivar (DB) of *L. chinensis* (Liu et al., 2026). Therefore, the HS cultivar was defined as a relatively saline-alkali-tolerant *L. chinensis*, while the DB cultivar was defined as a relatively saline-alkali-sensitive *L. chinensis*. Both *L. chinensis* cultivars (HS and DB) were provided by the Grassland Research Institute of the Chinese Academy of Agricultural Sciences. The soils used in this study were collected from the non-saline-alkali soil (0~30 cm depth) at the forage grass experimental station of Shihezi University in Xinjiang (44°20′59′′ N, 85°57′32′′ E) and the severely saline-alkali soil (0~30 cm depth) in the 147th Regiment of Shihezi City, Xinjiang (44°36′23′′ N, 85°1′0′′ E). After collection, the soils were fully homogenized, sieved through a 5 mm mesh, and stored for subsequent experiments. Non-saline-alkali soil and severe saline-alkali soil were mixed according to the weight ratio to conduct the compound mixed saline-alkali stress experiment. According to the total salt content, three treatments were established: non-saline-alkali soil (CK), moderate saline-alkali soil (MS), and severe saline-alkali soil (ES) (referring to the salinization classification standard of the Second National Soil Survey of China) (Li, 2010). The salt contents of the three treatments were 0.1%, 0.6%, and 1.2% respectively, and their ion composition included Cl^-^, HCO_3_^-^, SO_4_²^-^, Ca²^+^, Mg²^+^, K^+^, Na^+^, etc. (**Table 1**), which was designed to simulate the natural saline-alkali environment. The uniformly mixed saline-alkali soil was put into flowerpots with a diameter and depth of 18 cm, with 3 kg of soil per pot.

**Table 1:** Soil physical and chemical properties.

Treatment	Total salt (%)	pH	Electrical conductivity (dS/m)	Cl^-^ (g/kg)	HCO_3_^-^ (g/kg)	SO_4_^2-^ (g/kg)	Ca^2+^ (g/kg)	Mg^2+^ (g/kg)	K^+^ (g/kg)	Na^+^ (g/kg)
CK	0.1	7.42	0.71	0.16	0.08	1.23	0.04	0.01	0.01	0.07
MS	0.6	7.87	2.84	0.48	0.17	6.54	0.08	0.02	0.01	0.23
ES	1.2	8.34	6.99	1.03	0.32	19.91	0.12	0.03	0.02	1.31

CK, non-saline-alkali soil; MS, moderate saline-alkali soil; ES, severe saline-alkali soil. Owing to the extremely low content of CO_3_^2-^ in the tested soil, the data of this indicator were not statistically analyzed in this study.

*L. chinensis* seeds were surface-sterilized with 75% ethanol for 1 min, rinsed thoroughly with deionized water, and then placed on sterile gauze for germination. Germination was conducted in the dark at a constant temperature (25°C) with constant humidity for 24 h. The germinated seeds were then transplanted into plug trays. In May 2024, uniform three-leaf-stage seedlings with consistent morphology were transplanted into the pots filled with the prepared soils, with 10 seedlings per pot. Each treatment included 15 pots, resulting in a total of 90 pots for the entire experiment. All *L. chinensis* plants were grown in a greenhouse at 25°C with a 16 h/8 h (light/dark) photoperiod, The experiment lasted for one year, with watering performed every three days to maintain soil moisture. Since the growth periods of HS and DB *L. chinensis* were consistent, the agronomic traits and photosynthetic parameters of plants in each group were determined during the regreening stage of *L. chinensis* (later stage) in April 2025. Meanwhile, the roots and top 2–3 fully expanded leaves of *L. chinensis* were collected, quickly frozen in liquid nitrogen, and stored at -80°C for subsequent determinations of physiological and biochemical indicators and omics sequencing.

### 2.2 Determination of agronomic traits

*L. chinensis* plants with uniform growth were randomly selected from each treatment group to determine their morphological and biomass indices. Plant height and root length were measured using a steel tape (cm), and the number of fully expanded leaves per plant was recorded. The aboveground parts of *L. chinensis* were subsequently excised from the soil surface. Immediately after excision, the samples were placed in an oven at 105 °C for 30 min to inactivatee enzymes. Afterward, the oven temperature was adjusted to 65 °C, and the samples were dried to a constant weight. The dried aboveground samples from 5 individual plants per treatment were pooled and weighed, and the resulting weight was recorded as the aboveground biomass.

The second fully expanded leaf from the top of *L. chinensis* plants with uniform growth was selected for photosynthetic measurement (Yan et al., 2022). On 28 April 2025, from 11:00 to 13:00, a Li-6400XT portable photosynthesis system (Li-COR, Lincoln, NE, USA) was used to measure transpiration rate (*Tr*), net photosynthetic rate (*Pn*), intercellular CO_2_ concentration (*Ci*), and stomatal conductance (*Gs*). The photosynthetically active radiation (PAR) was maintained at 600~800 µmol·m^-^²·s^-^¹ throughout the measurements.

A 0.2 g fresh *L. chinensis* leaf sample was weighed and placed in a mortar. Subsequently, 5 mL of 95% ethanol was added, and the sample was ground into a homogeneous paste. The entire homogenate was transferred and filtered into a brown volumetric flask, after which 95% ethanol was added to bring the volume up to 25 mL. A spectrophotometer (Model T6, Persee, Beijing, China) was used to measure the absorbance of the samples at 470 nm, 649 nm, and 665 nm. The contents of chlorophyll a (Chl a), chlorophyll b (Chl b), carotenoids (Car), and total chlorophyll (Total chl) were calculated using the formulas described in (Wu et al., 2025).

### 2.3 Determination of physiological characteristics

#### 2.3.1 Determination of malondialdehyde content

The malondialdehyde (MDA) content in *L. chinensis* leaves was determined using a kit (Nanjing Jiancheng Institute of Bioengineering). A 0.1 g fresh *L. chinensis* leaf sample was weighed, and extraction buffer was added at a weight-to-volume ratio of 1:9 (g/mL). The mixture was homogenized in an ice bath, followed by centrifugation at 10,000 rpm for 10 min at 4 °C. The supernatant was collected, and the reagents provided in the kit were added to it. Subsequently, the mixture was incubated in a boiling water bath at 95 °C for 20 min and then cooled under running water. The absorbance of each sample was measured at 530 nm, and the MDA content was calculated according to the kit instructions.

#### 2.3.2 Determination of hydrogen peroxide content

The hydrogen peroxide (H_2_O_2_) content in *L. chinensis* leaves was determined using a kit (Suzhou Keming Biotechnology Co., Ltd.). A 0.1 g fresh *L. chinensis* leaf sample was weighed, and 1 mL of extraction buffer was added to it. The mixture was homogenized in an ice bath, followed by centrifugation at 8,000 rpm for 10 min at 4 °C. The supernatant was collected, and the reagents provided in the kit were added to it. The absorbance of each sample was subsequently measured at 415 nm, and the H_2_O_2_ content was calculated according to the the kit instructions.

#### 2.3.3 Determination of antioxidant enzyme activity

The peroxidase (POD), catalase (CAT) and superoxide dismutase (SOD) activities in *L. chinensis* leaves were measured using kits (Suzhou Keming Biotechnology Co., Ltd.). A 0.1 g fresh *L. chinensis* leaf sample was weighed, and 1 mL of extraction buffer was added to it. This mixture was homogenized in an ice bath and centrifuged at 8,000 rpm for 10min at 4 °C, after which the reagents were added according to the instructions of the kit. The absorbance of each sample was measured at 470 nm (POD), 450 nm (SOD) and 240 nm (CAT). The corresponding POD, CAT, and SOD activities were calculated according to the kit instructions.

#### 2.3.4 Determination of osmotic regulatory substances

The contents of proline (Pro), soluble protein (SP) and soluble sugar (SS) in *L. chinensis* leaves were determined using kits (Suzhou Keming Biotechnology Co., Ltd.). A 0.1 g fresh *L. chinensis* leaf sample was weighed, and 1 mL of extract was added and homogenized in an ice bath. The absorbance was measured at 520 nm (Pro) and 620 nm (SS and SP), and the corresponding contents of Pro, SP, and SS were calculated according to the kit instructions.

#### 2.3.5 Determination of ion content

The K^+^ and Na^+^ concentrations in the leaves and roots of *L. chinensis* were determined according to the methods described by (Su et al., 2025). First, 0.1 g of the dry sample was weighed into a 150 mL conical flask, 5 mL of concentrated sulfuric acid was added, and the mixture was left to stand overnight. The conical flask was placed on an electric hot plate and heated slowly and uniformly until dense white fumes were generated. Subsequently, 1 mL of 30% H_2_O_2_ was slowly added around the bottle mouth, and the mixture was heated and shaken; this process was repeated 2–3 times until the solution was clear. The solution was filtered into a 50 mL volumetric flask and diluted to the scale line with ultrapure water. K^+^ and Na^+^ concentrations in leaves and roots were subsequently determined using an atomic absorption spectrophotometer (240 DUO, Agilent, Santa Clara, CA, USA).

#### 2.3.6 Determination of root vitality

The root vitality of *L. chinensis* was determined according to the methods described by (Higa et al., 2010). First, a 0.1 g fresh root tip sample was placed into a 10 mL beaker, and 2.5 mL of 4 g/L 2,3,5-triphenyltetrazolium salt (TTC) solution and 2.5 mL of phosphate buffer were added, completely submerging the sample. The samples were kept in a dark incubator at 37 °C for 3 h, after which 1 mL of 1 mol/L sulfuric acid was added to terminate the reaction. At the same time, a control group was established. The same mass of root tips was placed into a 10 mL beaker, and 1 mL of 1 mol/L sulfuric acid was added to kill the roots; the remaining steps were the same as those for the experimental group samples. The samples were removed, dried and ground with 10 mL of ethyl acetate. The extracts were collected and the absorbance of the extracts was measured at 485 nm.

TTC reduction strength [mg/(g·h)]=m/(m_0_·t)

Where m represents the TTC reduction (mg) obtained through the standard curve, M_0_ is the root sample mass (g), and t is the reaction time (h).

### 2.4 Collection and extraction of root exudates

The complete roots of *L. chinensis* were collected, and damage to the root epidermis and root hairs was avoided throughout the entire operation. Subsequently, the root surfaces were gently rinsed with sterile deionized water, and rinsing was repeated until no visible soil particles adhered to the root surfaces, ensuring no soil residue on the root surfaces and no damage to the root structure. After rinsing, the roots were transferred to a sterile glass Erlenmeyer flask containing 200 mL of sterile deionized water. The flask bottle wall was sealed with sterile aluminum foil, and the solution was collected every 2 hours in a dark environment (Cao et al., 2024). After multiple collections under the same conditions (6 times), the collected solutions were thoroughly mixed. The mixed collected solution was vacuum-filtered through a 0.22 μm sterile filter to remove suspended impurities and a small amount of shed root cells, and biological replicates were set up for each treatment. Subsequently, the filtrate was freeze-dried to obtain root exudate dry powder, which was hermetically sealed and stored in an ultra-low temperature freezer at -80°C for subsequent detection and analysis. The freeze-dried root exudate samples were sent to Biozeron Co., Ltd. (Shanghai, China) for root exudate extraction. Ultra-performance liquid chromatography-tandem mass spectrometry (UHPLC-MS/MS) was performed using a combination of a Vanquish UHPLC system (Thermo Fisher, Germany) with an Orbitrap Q Exactive™ HF mass spectrometer (Thermo Fisher, Germany).

### 2.5 Metabolomic analysis, metabolite extraction and profiling

Metabolite extraction and quantification were performed according to previously described methods (Cao et al., 2024; Wang et al., 2025). Lyophilized samples (100 mg) were extracted with 1.2 mL of 80% methanol at 4°C. After centrifugation at 12,000 rpm for 15 min, the supernatants were analyzed by UPLC-MS/MS. The mobile phase consisted of ultrapure water containing 0.1% formic acid (eluent A) and acetonitrile containing 0.1% formic acid (eluent B). The gradient elution program was set as follows: an initial proportion of 5% B, which was linearly increased to 95% B within 9 min, maintained for 1 min, then rapidly returned to 5% B in 0.1 min, followed by re-equilibration for 4.9 min (total run time: 15 min). The flow rate was 0.35 mL/min, and the injection volume was 4 μL. Differentially accumulated metabolites (DAMs) were screened based on the criteria of variable importance in the projection (VIP) > 1, *P* < 0.05 (Student’s t-test), and |log2(FC)| ≥ 1. Metabolic pathway annotation and enrichment analysis of DAMs were performed using the KEGG database to identify key involved metabolic pathways, with KEGG enrichment analysis conducted at a threshold of *P* < 0.05.

### 2.6 Data analysis

The data were organized using Excel 2019, and t-test and two-way analysis of variance (two-way ANOVA) were performed using SPSS 20.0 (IBM, Armonk, NY, USA). Multiple comparisons were conducted via Tukey’s test (*P* < 0.05), and all data were expressed as mean ± standard deviation. Growth and photosynthetic physiology bar charts were plotted with Origin 2021 and Lingbo MicroClass (http://www.cloud.biomicroclass.com/CloudPlatform).

## 3 Results

### 3.1 Effects of saline-alkali stress on the agronomic traits of *L. chinensis*

**Figure 1:**
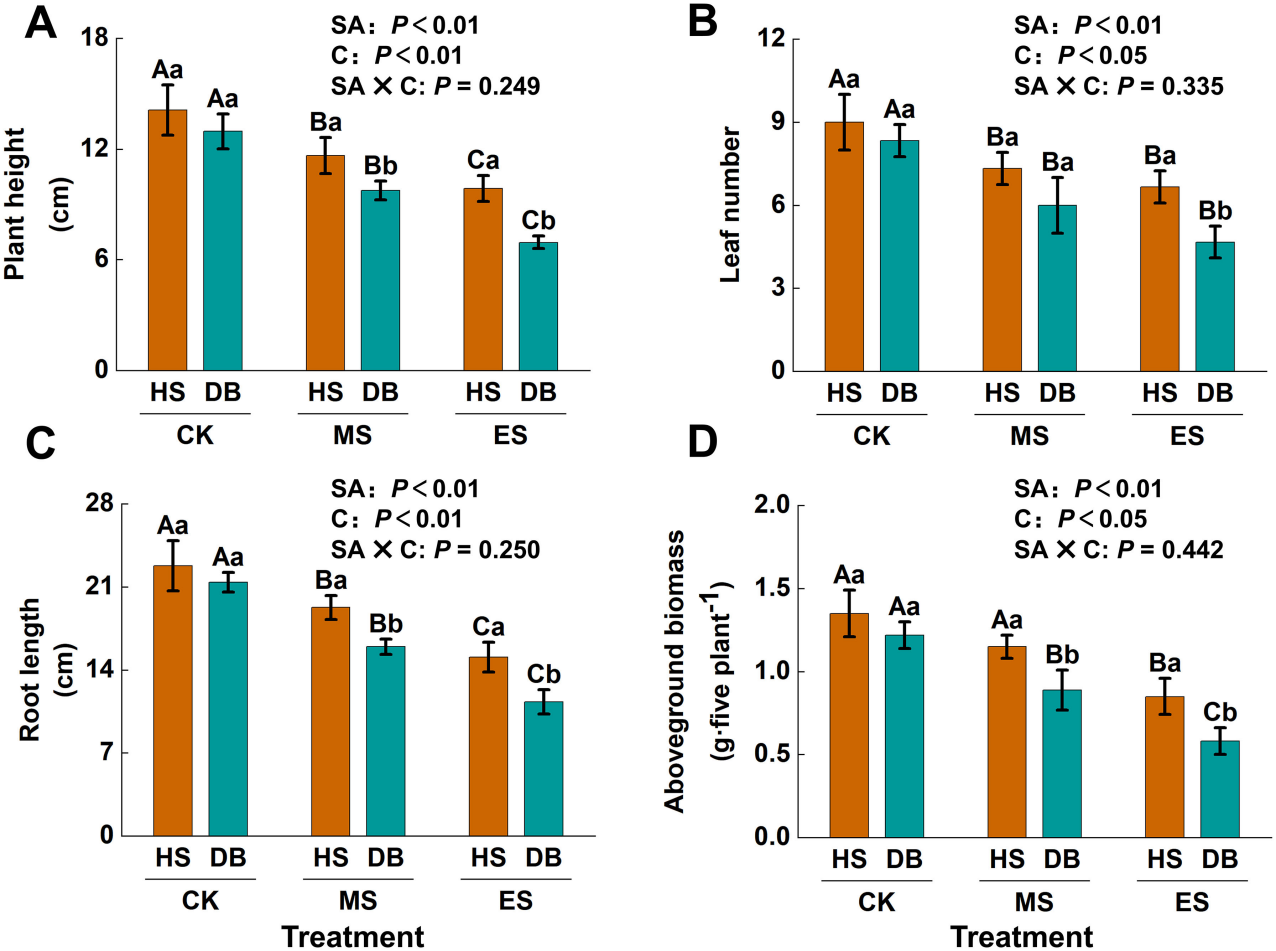
Effects of saline-alkali stress on the agronomic traits of *L. chinensis*. **(A)** Plant height, **(B)** Leaf number, **(C)** Root length, **(D)** Aboveground biomass. SA, saline-alkali stress; C, cultivar; CK, non-saline-alkali soil; MS, moderate saline-alkali soil; ES,severe saline-alkali soil. HS, relatively saline-alkali-tolerant cultivar ‘Huise’, DB, relatively saline-alkali-sensitive cultivar ‘Dongbei’. The data are presented as the mean ± standard deviation (n = 3). Different lowercase letters indicate significant differences among different *L.chinensis* cultivars under the same saline-alkali stress treatment (*P* < 0.05), whereas different uppercase letters indicate significant differences among the same *L.chinensis* cultivars under different saline-alkali stress treatments (*P* < 0.05).

Saline-alkali stress (SA) and cultivar (C) had a significant effected on the plant height, leaf number, root length, and aboveground biomass. Specifically, with the increase in saline-alkali stress intensity, the morphological indicators of the HS and DB *L. chinensis* cultivars showed an overall decreasing trend (**Figure 1**). Compared with those under the CK treatment, the plant height, leaf number, root length, and aboveground biomass of HS *L. chinensis* under the ES treatment decreased by 30.01%, 25.93%, 33.76%, and 37.04%, respectively; in contrast, those of DB *L. chinensis* under the ES treatment decreased by 46.35%, 44.00%, 47.15%, and 52.45%, respectively. Under both the MS and ES treatments, compared with those of DB *L. chinensis*, the plant height, root length, and aboveground biomass of HS *L. chinensis* were significantly higher.

### 3.2 Effects of saline-alkali stress on the photosynthetic traits of *L. chinensis*

**Figure 2:**
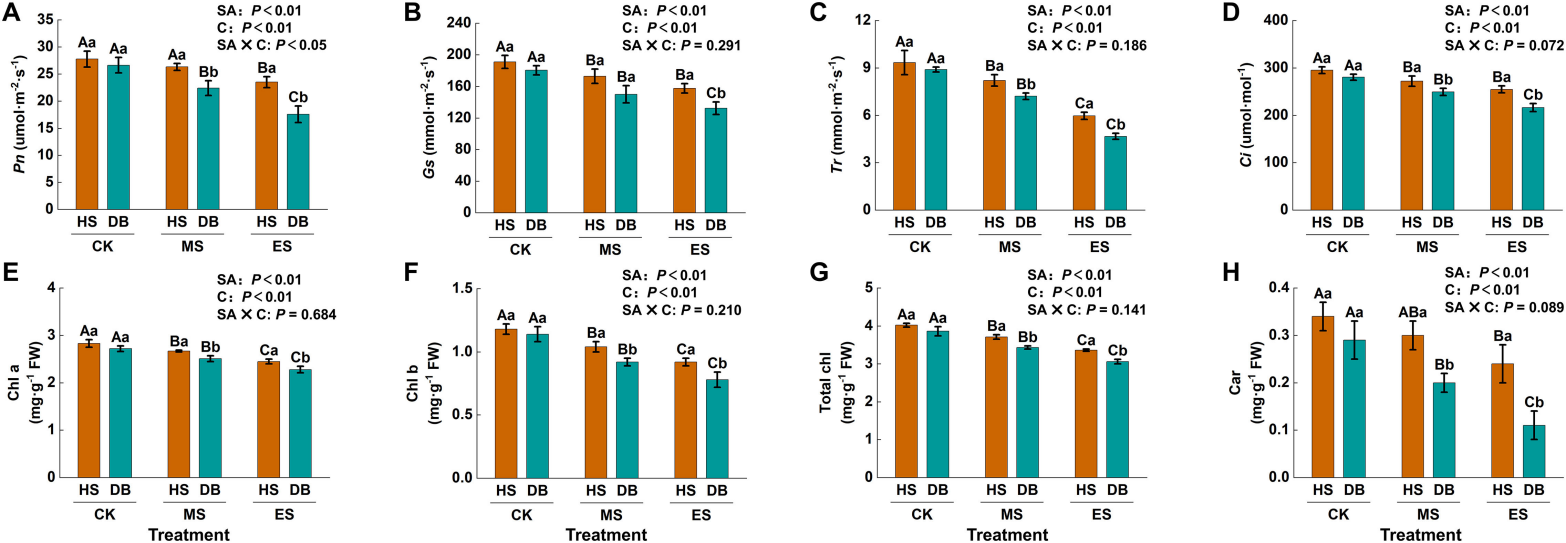
Effects of saline-alkali stress on the photosynthetic characteristics of *L. chinensis*. **(A)** Net photosynthetic rate (*Pn*), **(B)** Stomatal conductance (*Gs*), **(C)** Transpiration rate (*Tr*), **(D)** Intercellular CO_2_ concentration (*Ci*), **(E)** Chlorophyll a (Chl a), **(F)** Chlorophyll b (Chl b), **(G)** Total chlorophyll (Total chl), **(H)** Carotenoid (Car). SA, saline-alkali stress. C, cultivar. CK, non-saline-alkali soil; MS, moderate saline-alkali soil; ES, severe saline-alkali soil. HS, relatively saline-alkali-tolerant cultivar ‘Huise’; DB, relatively saline-alkali-sensitive cultivar ‘Dongbei’. The data are presented as the mean ± standard deviation (n = 3). Different lowercase letters indicate significant differences among different *L.chinensis* cultivars under the same saline-alkali stress treatment (*P* < 0.05), whereas different uppercase letters indicate significant differences among the same *L.chinensis* cultivars under different saline-alkali stress treatments (*P* < 0.05).

Saline-alkali stress and cultivar had significant effected on the photosynthetic parameters and photosynthetic pigment contents of *L. chinensis*, and their interaction significantly inhibited the *Pn* of *L. chinensis*. Specifically, with the increase of saline-alkali stress intensity, the photosynthetic parameters (**Figures 2A–D**) and photosynthetic pigments (**Figures 2E–H**) of HS and DB *L. chinensis* cultivars showed a decreasing trend. Compared with those under the CK treatment, the *Pn*, *Ci*, *Gs*, and *Tr* of HS *L. chinensis* under the ES treatment decreased by 15.31%, 13.65%, 17.55%, and 36.10%, respectively; in contrast, those of DB *L. chinensis* under the ES treatment decreased decreased by 33.94%, 22.79%, 26.67%, and 47.57%, respectively. In terms of photosynthetic pigments, compared with those under the CK treatment, the Chl a, Chl b, Total chl, and Car contents in the HS *L. chinensis* treatment under the ES treatment decreased by 13.64%, 22.54%, 16.26%, and 29.41%, respectively, in contrast, those in the DB *L. chinensis* treatment under the ES treatment decreased by 16.27%, 31.38%, 20.72%, and 63.22%. Moreover, the *Pn*, *Ci*, *Gs*, *Tr*, Chl a, Chl b, Total chl, and Car of DB *L. chinensis* were significantly lower than those of HS *L. chinensis*.

### 3.3 Effects of saline-alkali stress on physiological traits of *L. chinensis*

**Figure 3:**
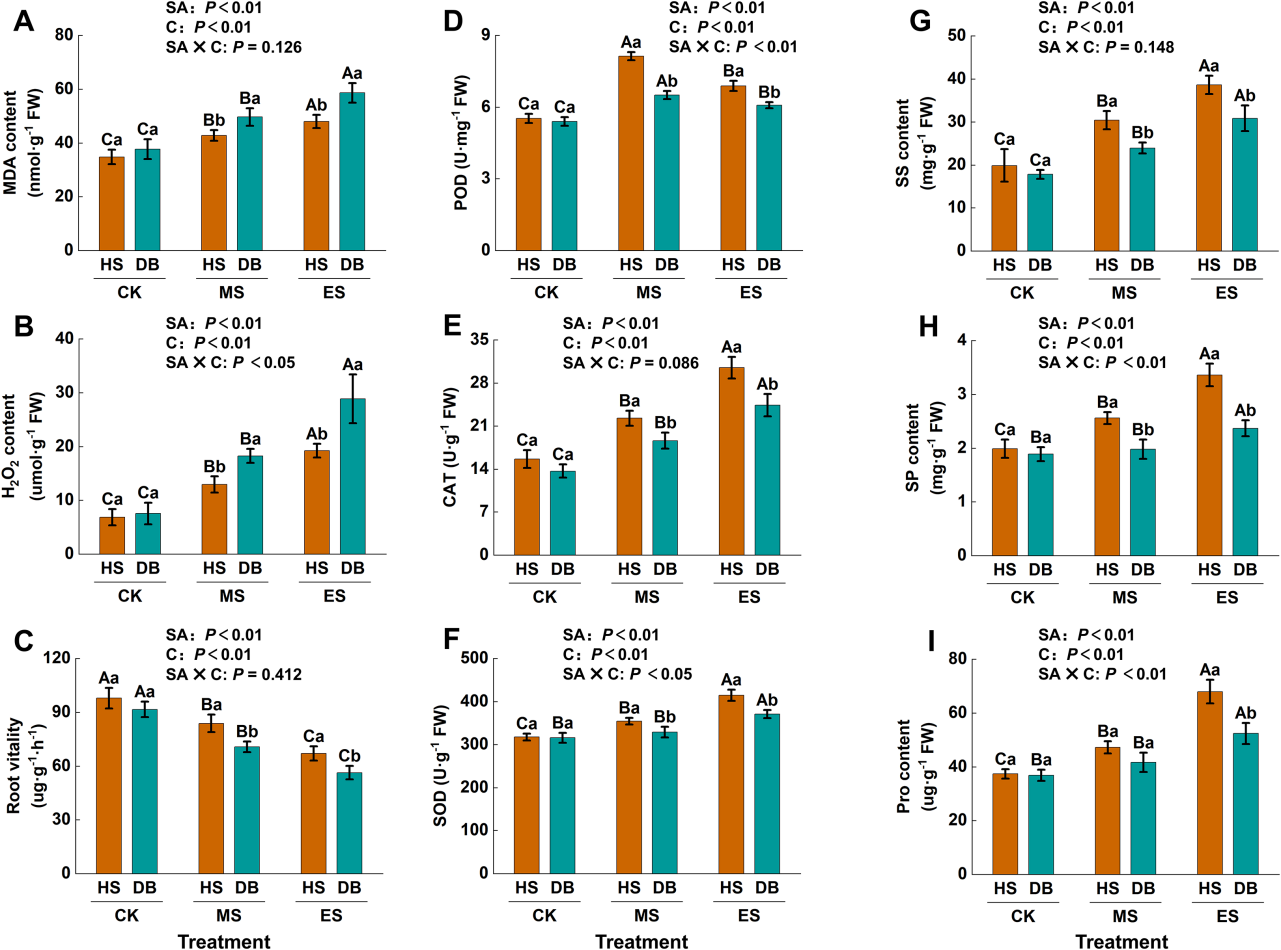
Effects of saline-alkali stress on the physiological traits of *L. chinensis*. **(A)** Malondialdehyde (MDA), **(B)** Hydrogen peroxide (H_2_O_2_), **(C)** Root vitality, **(D)** Peroxidase (POD), **(E)** Catalase (CAT), **(F)** Superoxide dismutase (SOD), **(G)** Soluble sugar (SS), **(H)** Soluble protein (SP), **(I)** Proline (Pro). SA: saline-alkali stress. C, cultivar. CK, non-saline-alkali soi; MS, moderate saline-alkali soil; ES, severe saline-alkali soil. HS, relatively saline-alkali-tolerant cultivar ‘Huise’; DB, relatively saline-alkali-sensitive cultivar ‘Dongbei’. The data are presented as the mean ± standard deviation (n = 3). Different lowercase letters indicate significant differences among different *L.chinensis* cultivars under the same saline-alkali stress treatment (*P* < 0.05), whereas different uppercase letters indicate significant differences among the same *L.chinensis* cultivars under different saline-alkali stress treatments (*P* < 0.05).

Saline-alkali stress and cultivar significantly affected the contents of MDA, H_2_O_2_, Pro, SP, and SS, the activities of POD, CAT, and SOD, as well as root vitality in *L. chinensis*. Saline-alkali stress and cultivar promoted the accumulation of MDA and H_2_O_2_, increased antioxidant enzyme activities, and markedly inhibited root vitality. Their interaction significantly influenced the contents of H_2_O_2_, Pro, and SP, and the activities of POD and SOD in *L. chinensis*. With increasing saline-alkali stress intensity, the MDA and H_2_O_2_ contents of HS and DB *L. chinensis* peaked under the ES treatment (**Figures 3A, B**), and these values were significantly greater than those under the other saline-alkali stress treatments. The root vitality of *L. chinensis* tended to gradually decrease. Compared with those in the CK treatment, the root vitality in the HS and DB *L. chinensis* (**Figure 3C**) decreased by 31.44% and 38.50%, respectively, in the ES treatment; these decreases were significantly lower than those in the other saline-alkali stress treatments. In contrast, the contents of Pro, SP, and SS, as well as the activities of POD, CAT, and SOD, tended to increase in HS and DB *L. chinensis* with increasing saline-alkali stress intensity (**Figures 3D–I**). The contents of Pro, SP and SS, as well as the activities of CAT and SOD, reached a maximum under the ES treatment, while the POD activity reached a maximum under the MS treatment; all of these indices were significantly greater than those under the other saline-alkali stress treatments. Under both the MS and ES treatments, the contents of Pro, SP and SS, as well as the activities of POD, CAT, and SOD, were significantly greater in HS *L. chinensis* than in DB *L. chinensis*.

### 3.4 Effects of saline-alkali stress on the ion content of *L. chinensis*

**Figure 4:**
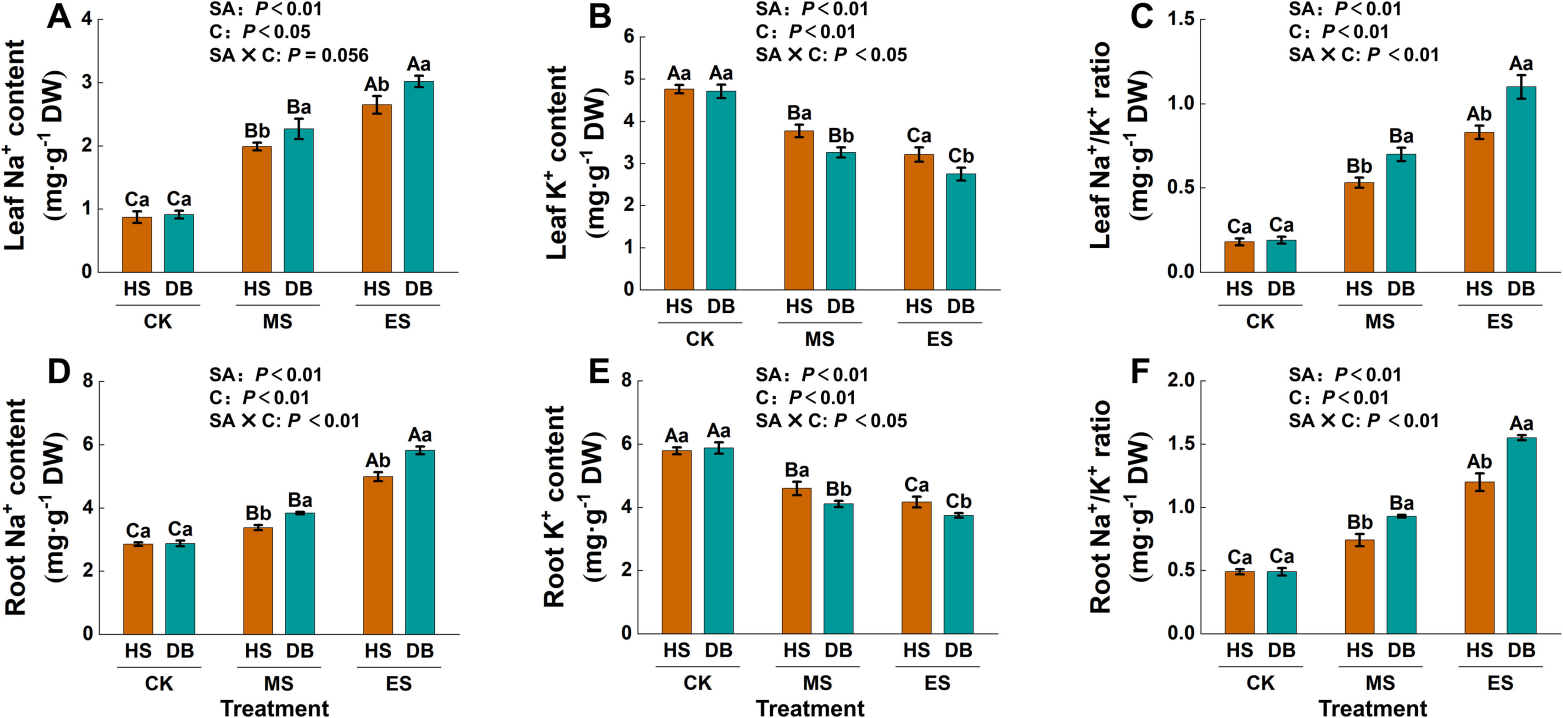
Effects of saline-alkali stress on the ion content of *L. chinensis*. **(A)** Leaf Na^+^ content, **(B)** Leaf K^+^ content, **(C)** Leaf Na^+^/K^+^ ratio, **(D)** Root Na^+^ content, **(E)** Root K^+^ content, **(F)** Root Na^+^/K^+^ ratio. SA, saline-alkali stress. C, cultivar. CK, non-saline-alkali soil; MS, moderate saline-alkali soil; ES, severe saline-alkali soil. HS, relatively saline-alkali-tolerant cultivar ‘Huise’; DB, relatively saline-alkali-sensitive cultivar ‘Dongbei’. The data are presented as the mean ± standard deviation (n = 3). Different lowercase letters indicate significant differences among different *L.chinensis* cultivars under the same saline-alkali stress treatment (*P* < 0.05), whereas different uppercase letters indicate significant differences among the same *L.chinensis* cultivars under different saline-alkali stress treatments (*P* < 0.05).

Saline-alkali stress and cultivars significantly affected the Na^+^, K^+^ contents and Na^+^/K^+^ ratio in the leaves and roots of *L. chinensis*. Their interaction significantly influenced the K^+^ content and Na^+^/K^+^ ratio in the leaves, as well as the Na^+^, K^+^ contents and Na^+^/K^+^ ratio in the roots of *L. chinensis*. With increasing saline-alkali stress intensity, the Na^+^ content and Na^+^/K^+^ ratio in the leaves and roots of *L. chinensis* tended to increase. The Na^+^ content and Na^+^/K^+^ ratio in leaves and roots peaked under the ES treatment, and these values were significantly greater than those under the other saline-alkali stress treatments (**Figure 4**). In contrast, the K^+^ content in leaves and roots was the lowest under the ES treatment, and these value was significantly lower than that under the other saline-alkali stress treatments. Under both the MS and ES treatments, the Na^+^ content and Na^+^/K^+^ ratio in the roots and leaves of HS *L. chinensis* were significantly lower than those in DB *L. chinensis*, while the K^+^ content was significantly greater than that in DB *L. chinensis*.

### 3.5 Effects of saline-alkali stress on the root exudates of *L. chinensis*

#### 3.5.1 Composition of root exudates and pattern recognition analysis

**Figure 5:**
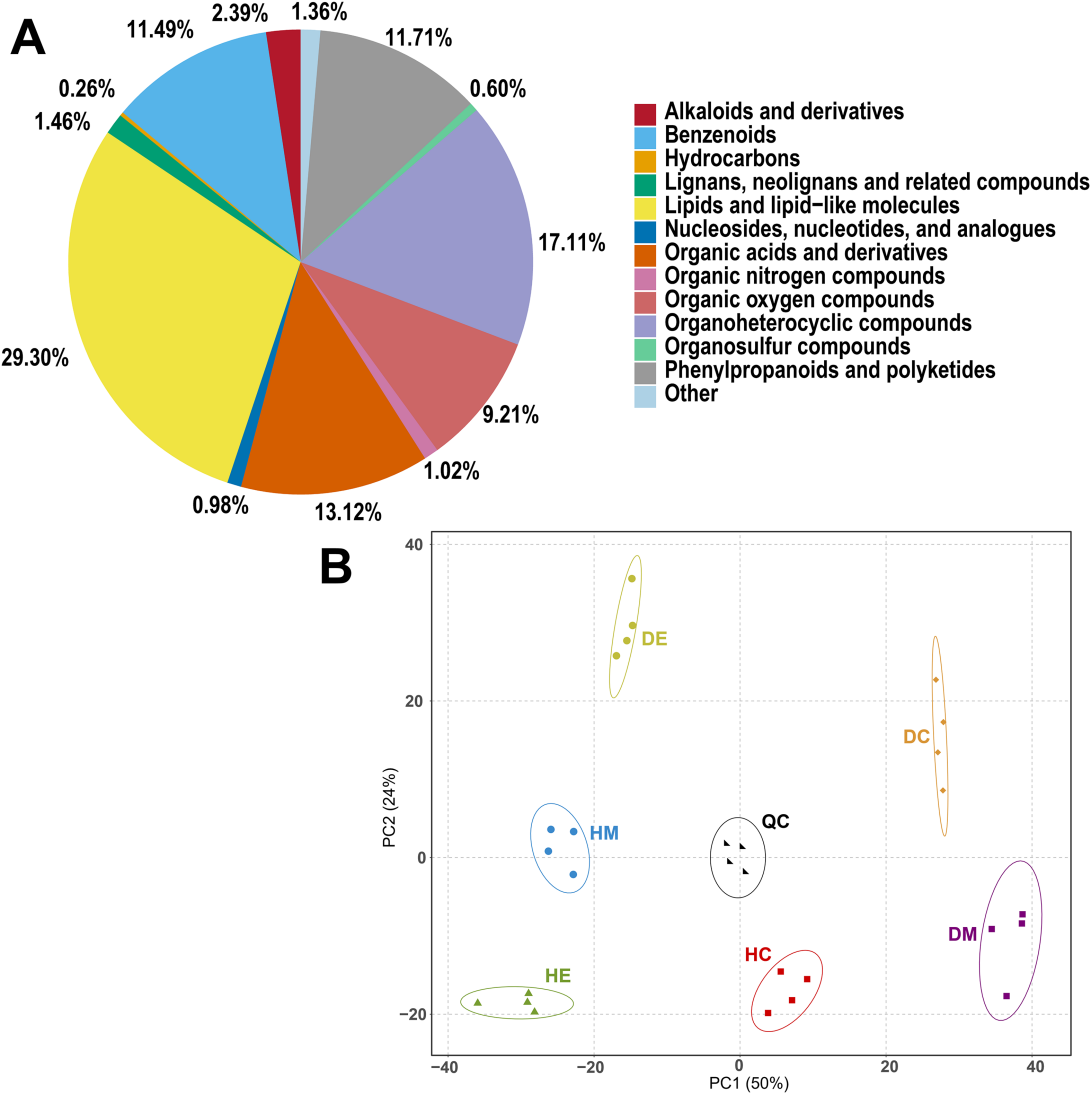
Analysis of the composition and pattern recognition of root exudates of *L. chinensis* under saline-alkali stress. **(A)** Pie charts of metabolite classification for all groups, **(B)** Principal coordinates analysis of metabolites in all groups. HC, HM, and HE represent the relatively saline-alkali-tolerant cultivar ‘Huise’ (HS) under CK, MS, and ES treatments, respectively; DC, DM, and DE represent the relatively saline-alkali-sensitive cultivar ‘Dongbei’ (DB) under CK, MS, and ES treatments, respectively.

Based on the UPLC-MS/MS detection platform and metabolic database, a total of 5014 metabolites were detected (**Figure 5A**). The chemical structures of the detected compounds were classified into the following categories: 120 alkaloids and derivatives (2.39%), 576 benzenes (11.49%), 13 hydrocarbons (0.26%), 73 lignans, neolignans and related compounds (1.46%), 1469 lipids and lipid-like molecules (29.30%), 49 nucleosides, nucleotides and analogues (0.98%), 658 organic acids and derivatives (13.12%), 51 organic nitrogen compounds (1.02%), 462 organic oxygen compounds (9.21%), 858 organoheterocyclic compounds (17.11%), 30 organosulfur compounds (0.60%), 587 phenylpropanoids and polyketides (11.71%), and 68 other metabolites (1.36%).

Principal coordinate analysis (PCoA) was performed on the root exudate metabolite profiles of different *L. chinensis* treatment groups to evaluate the differences between groups and the homogeneity of intra-samples. The PCoA results revealed that PC1 explained the largest proportion of the total variation in root exudate metabolites (50%), followed by PC2, which explained 24% of the total variation (**Figure 5B**). Samples from the same treatment group closely clustered together, indicating that all the treatments had good repeatability with small intra-samples differences. Furthermore, the different treatments clearly were clearly separated from each other, suggesting significant differences between the treatments. HM and DM were separated along the PC1 direction, while HC and DC, HE and DE were also distinctly separated along the PC2 direction, among which the DE group was the most distinct from the other groups. The PCoA results could effectively distinguish the metabolic characteristics of the two *L. chinensis* cultivars under different saline-alkali intensities, indicating that saline-alkali treatments and cultivars together affected the overall variation of metabolic components in root exudates.

#### 3.5.2 Screening of differentially accumulated metabolites

**Figure 6:**
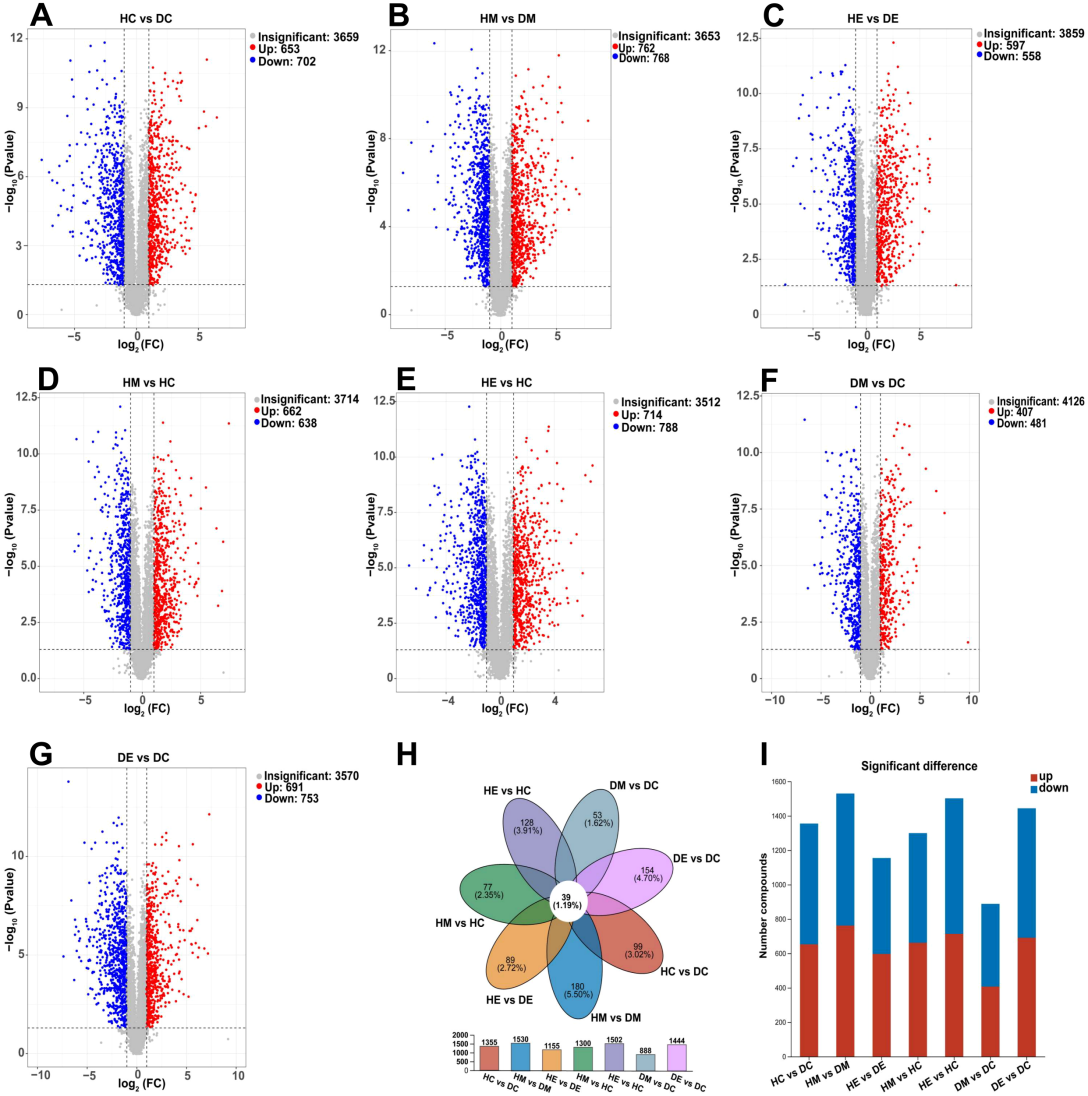
Screening and analysis of DAMs in *L. chinensis* under saline-alkali Stress. **(A–G)** Volcano plots of DAMs for comparisons of HC vs DC, HM vs DM, HE vs DE, HM vs HC, HE vs HC, DM vs DC, and DE vs DC, respectively. Venn diagram **(H)** and stacked bar chart **(I)** of the DAMs in all groups. Each dot represents one metabolite: blue dots represent metabolites with decreased abundance, red dots represent metabolites with increased abundance, and gray dots represent detected metabolites with no significant differences. HC, HM, and HE represent the relatively saline-alkali-tolerant cultivar ‘Huise’ (HS) under CK, MS, and ES treatments, respectively; DC, DM, and DE represent the relatively saline-alkali-sensitive cultivar ‘Dongbei’ (DB) under CK, MS, and ES treatments, respectively. For the comparison between different *L. chinensis* cultivars under the same saline-alkali stress, DB *L. chinensis* was used as the control; for the comparison of the same *L. chinensis* cultivar under different saline-alkali stress intensities, CK was used as the control.

Comparison of differentially accumulated metabolites (DAMs) between different *L. chinensis* cultivars under the same saline-alkali stress intensity: in the HC vs DC comparison, 653 DAMs were upregulated and 702 DAMs were downregulated (**Figure 6A**). In the HM vs DM comparison, 762 DAMs were upregulated and 768 DAMs were downregulated (**Figure 6B**). In the HE vs DE comparison, 597 DAMs were upregulated and 558 DAMs were downregulated (**Figure 6C**). Comparison of DAMs in *L. chinensis* under different saline-alkali stress intensities: in the HM vs HC comparison, 662 DAMs were upregulated and 638 DAMs were downregulated (**Figure 6D**). In the HE vs HC comparison, 714 DAMs were upregulated and 788 DAMs were downregulated (**Figure 6E**). In the DM vs DC comparison, 407 DAMs were upregulated and 481 DAMs were downregulated (**Figure 6F**). In the DE vs DC comparison, 691 DAMs were upregulated and 753 DAMs were downregulated (**Figure 6G**). The number of DAMs between different *L. chinensis* cultivars under the same saline-alkali stress intensity first increased and then decreased as stress intensity increased. For DAM numbers under different saline-alkali treatments, the total number of DAMs in DB *L. chinensis* was lower than that in HS *L. chinensis* (**Figure 6I**). A total of 39 common DAMs were shared across all comparison groups (**Figure 6H**). Overall, under identical stress conditions, inter-cultivar DAM numbers increased initially and then declined with rising saline-alkali intensity. Across different stress levels, the relatively saline-alkali-sensitive cultivar DB possessed fewer total DAMs than the relatively saline-alkali-tolerant cultivar HS, indicating obvious cultivar-specific differences in the metabolic remodeling of root exudates in *L. chinensis* under saline-alkali stress.

#### 3.5.3 Screening of DAM markers

**Figure 7:**
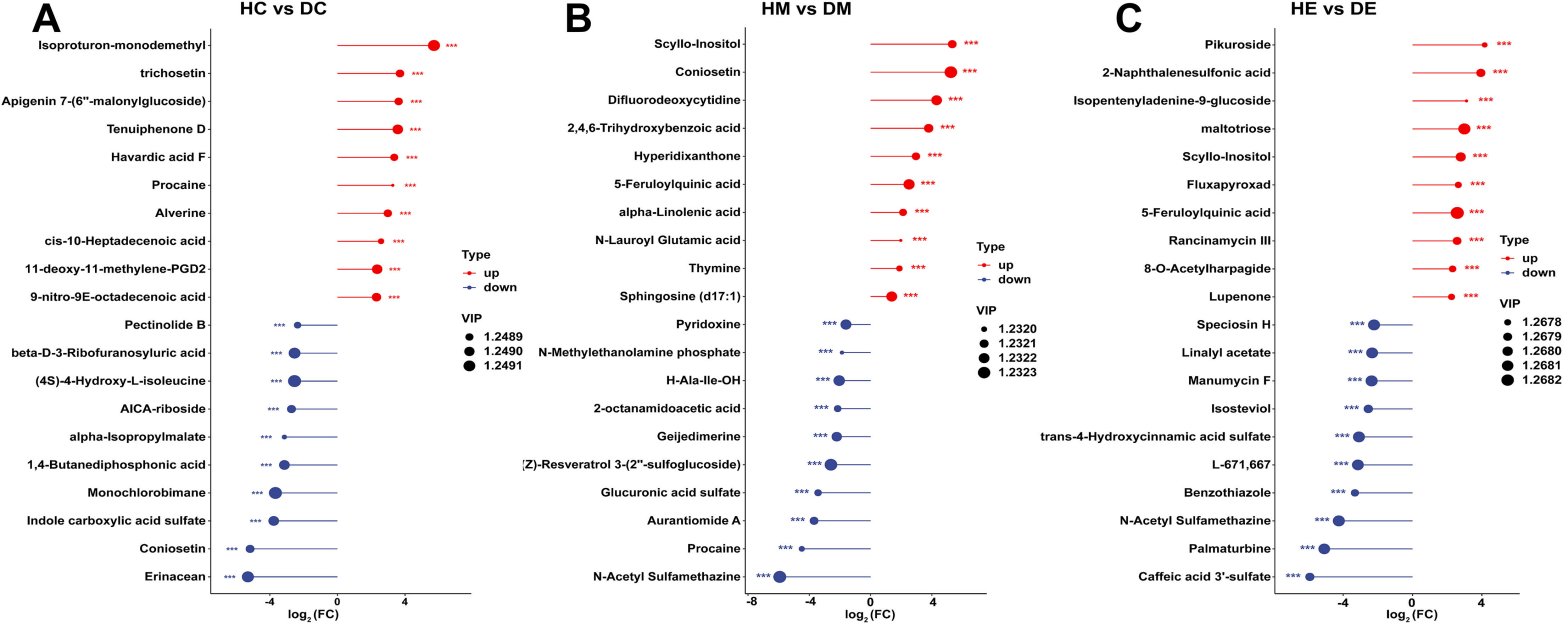
Screening of DAM markers in different *L.chinensis* under the same saline-alkali stress (FC-VIP analysis). **(A–C)** Lollipop plots of DAM markers for comparisons of HC vs DC, HM vs DM, and HE vs DE, respectively. HC, HM, and HE represent the relatively saline-alkali-tolerant cultivar ‘Huise’ (HS) under CK, MS, and ES treatments, respectively; DC, DM, and DE represent the relatively saline-alkali-sensitive cultivar ‘Dongbei’ (DB) under CK, MS, and ES treatments, respectively. For the comparison between different *L. chinensis* cultivars under the same saline-alkali stress, DB *L. chinensis* was used as the control.

**Figure 8:**
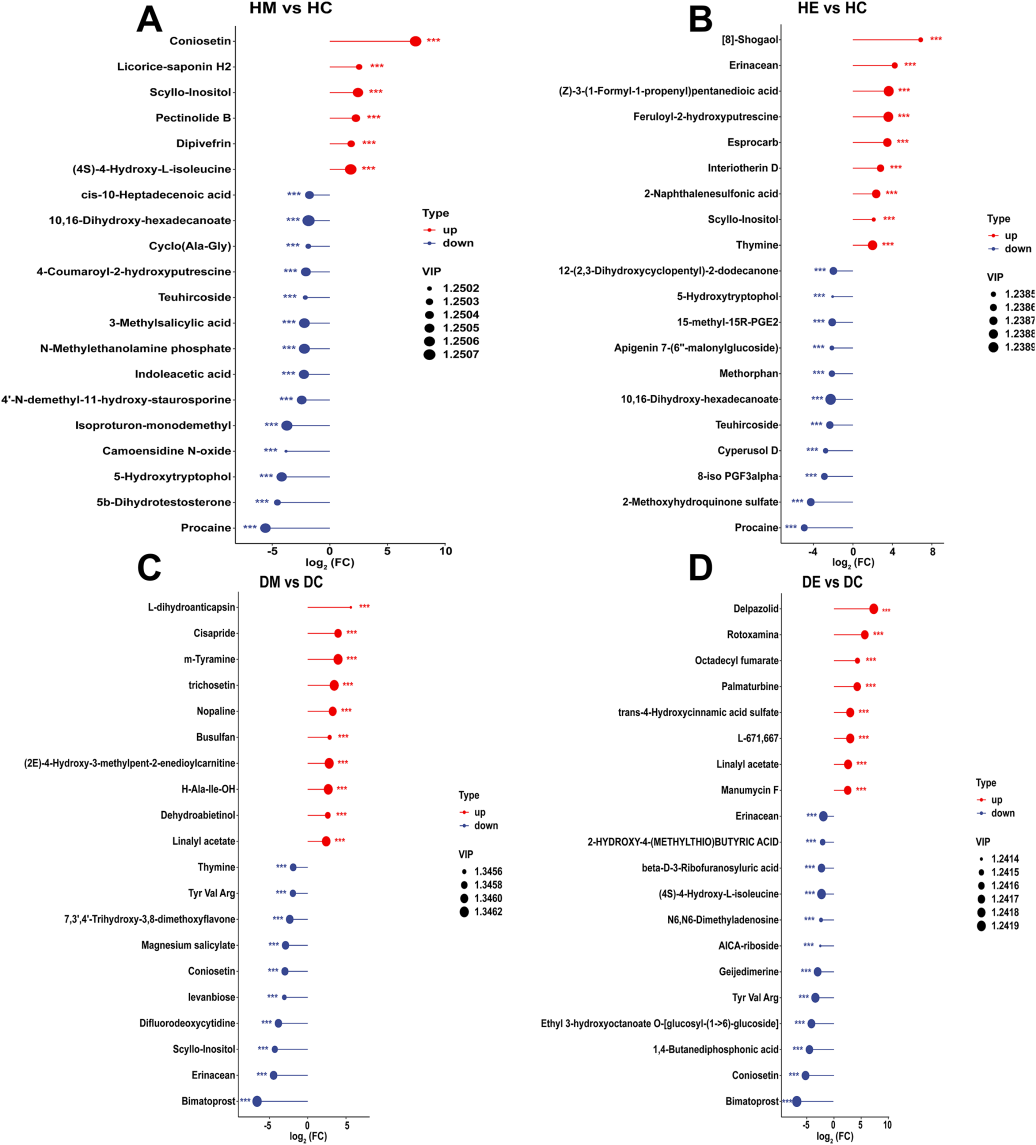
Screening of DAM markers of *L.chinensis* under different saline-alkali stresses (FC-VIP analysis). **(A–D)** Lollipop plots of DAM markers for comparisons of HM vs HC, HE vs HC, DM vs DC, and DE vs DC, respectively. HC, HM, and HE represent the relatively saline-alkali-tolerant cultivar ‘Huise’ (HS) under CK, MS, and ES treatments, respectively; DC, DM, and DE represent the relatively saline-alkali-sensitive cultivar ‘Dongbei’ (DB) under CK, MS, and ES treatments, respectively. For the comparison of the same *L. chinensis* cultivar under different saline-alkali stress intensities, CK was used as the control.

To further explore the variation of DAMs among comparison groups, the top 30 DAMs with the highest VIP values were selected from each group for fold change analysis (**Figures 7**, **8**). In the HC vs DC comparison, the |log_2_FC| values of isoproturon-monodemethyl, erinacean, and coniosetin were greater than 5, with the highest value reaching 5.71 (**Figure 7A**). In the HM vs DM comparison, the |log_2_FC| values of N-acetyl sulfamethazine, scyllo-inositol, and coniosetin exceeded 5, with a maximum value of 5.93 (**Figure 7B**). In the HE vs DE comparison, the |log_2_FC| values of caffeic acid 3’-sulfate and palmaturbine were greater than 5, and the highest value was 5.92 (**Figure 7C**).

In the HM vs HC comparison, the |log_2_FC| values of coniosetin and procaine were greater than 5, with the peak value reaching 7.44 (**Figure 8A**). In the HE vs HC comparison, the |log_2_FC| values of [8]-shogaol and procaine were greater than 4.5, and the maximum value was 6.87 (**Figure 8B**). In the DM vs DC comparison, the |log_2_FC| values of bimatoprost and L-dihydroanticapsin exceeded 5, with the highest value being 6.62 (**Figure 8C**). In the DE vs DC comparison, the |log_2_FC| values of delpazolid, rotoxamina, bimatoprost, and coniosetin were greater than 5, and the highest value reached 7.33 (**Figure 8D**).

#### 3.5.4 KEGG annotation and enrichment analysis

**Figure 9:**
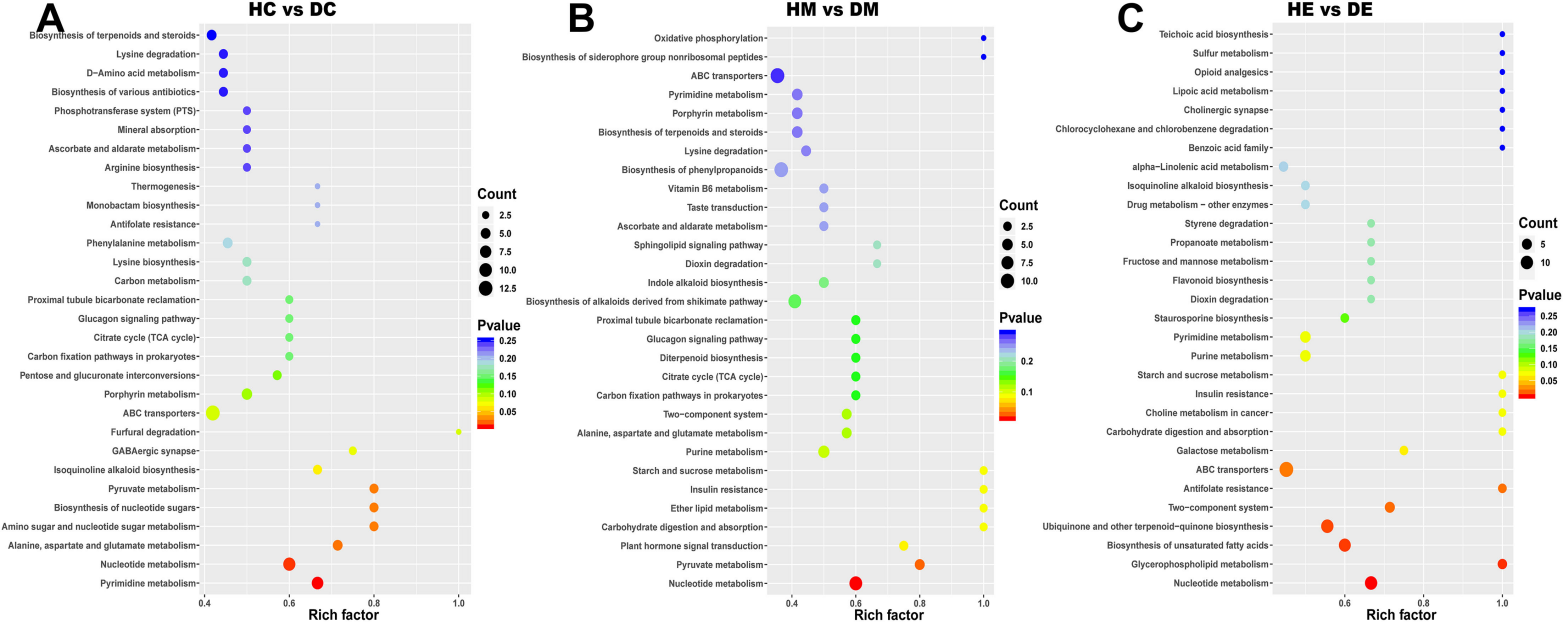
KEGG enrichment analysis of DAMs in different *L.chinensis* under the same saline-alkali stress. **(A–C)** KEGG pathway plots of DAMs for comparisons of HC vs DC, HM vs DM, and HE vs DE, respectively. Some enriched pathways such as the two-component system are evolutionarily conserved from prokaryotes and execute signal transduction functions in plants. The antifolate resistance pathway is matched due to shared core enzymatic reactions of folate metabolism. Universal KEGG database was used for metabolite annotation to ensure full coverage of detected metabolites. HC, HM, and HE represent the relatively saline-alkali-tolerant cultivar ‘Huise’ (HS) under CK, MS, and ES treatments, respectively; DC, DM, and DE represent the relatively saline-alkali-sensitive cultivar ‘Dongbei’ (DB) under CK, MS, and ES treatments, respectively. For the comparison between different *L. chinensis* cultivars under the same saline-alkali stress, DB *L. chinensis* was used as the control.

**Figure 10:**
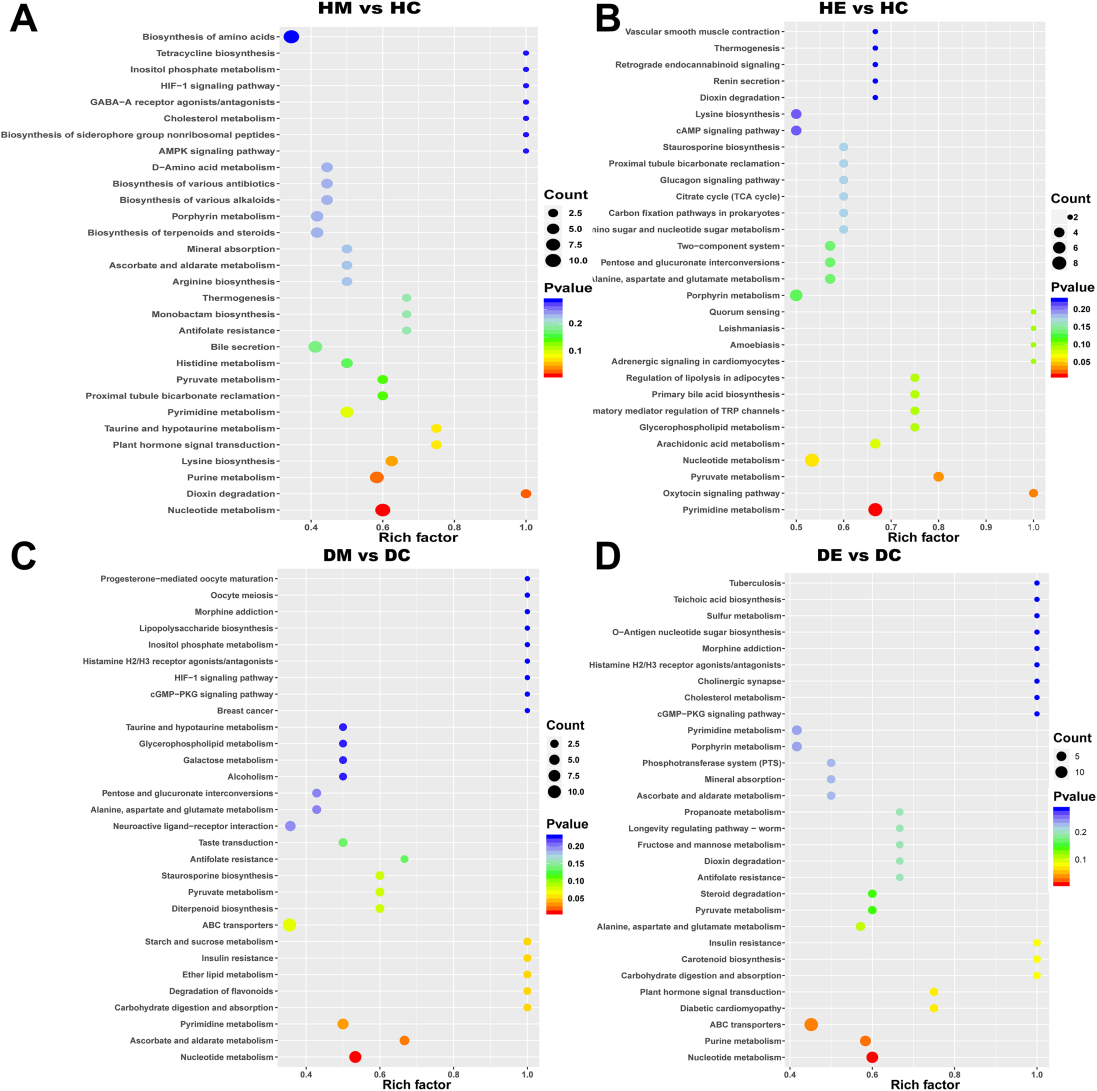
KEGG enrichment analysis of DAMs in different *L.chinensis* under the same saline-alkali stress. **(A–D)** KEGG pathway plots of DAMs for comparisons of HM vs HC, HE vs HC, DM vs DC, and DE vs DC, respectively. HC, HM, and HE represent the relatively saline-alkali-tolerant cultivar ‘Huise’ (HS) under CK, MS, and ES treatments, respectively; DC, DM, and DE represent the relatively saline-alkali-sensitive cultivar ‘Dongbei’ (DB) under CK, MS, and ES treatments, respectively. For the comparison of the same *L. chinensis* cultivar under different saline-alkali stress intensities, CK was used as the control.

To explore the functional diversity of DAMs in *L. chinensis* cultivars with different saline-alkali tolerances under saline-alkali stress, KEGG enrichment analysis was performed on DAMs from each comparison group to clarify their biological functions. The results identified the top 20 significantly enriched KEGG pathways in each group (**Figures 9**, **10**). In the HC vs DC comparison, 6 metabolic pathways were significantly enriched, including pyrimidine metabolism, nucleotide metabolism, alanine, aspartate and glutamate metabolism, pyruvate metabolism, amino sugar and nucleotide sugar metabolism, and biosynthesis of nucleotide sugars (**Figure 9A**). In the HM vs DM comparison, 2 metabolic pathways were significantly enriched: nucleotide metabolism and pyruvate metabolism (**Figure 9B**). In the HE vs DE comparison, 7 metabolic pathways were significantly enriched, namely nucleotide metabolism, glycerophospholipid metabolism, biosynthesis of unsaturated fatty acids, ubiquinone and other terpenoid-quinone biosynthesis, two-component system, antifolate resistance, and ABC transporters (**Figure 9C**).

In the HM vs HC comparison, 4 metabolic pathways were significantly enriched: nucleotide metabolism, dioxin degradation, purine metabolism, and lysine biosynthesis (**Figure 10A**). In the HE vs HC comparison, 3 metabolic pathways were significantly enriched: pyrimidine metabolism, oxytocin signaling pathway, and pyruvate metabolism (**Figure 10B**). In the DM vs DC comparison, 3 metabolic pathways were significantly enriched: nucleotide metabolism, ascorbate and aldarate metabolism, and pyrimidine metabolism (**Figure 10C**). In the DE vs DC comparison, 3 metabolic pathways were significantly enriched: nucleotide metabolism, purine metabolism, and ABC transporters (**Figure 10D**).

#### 3.5.5 Comprehensive set of metabolic pathways related to saline-alkali tolerance of *L. chinensis* under saline-alkali stress

**Figure 11:**
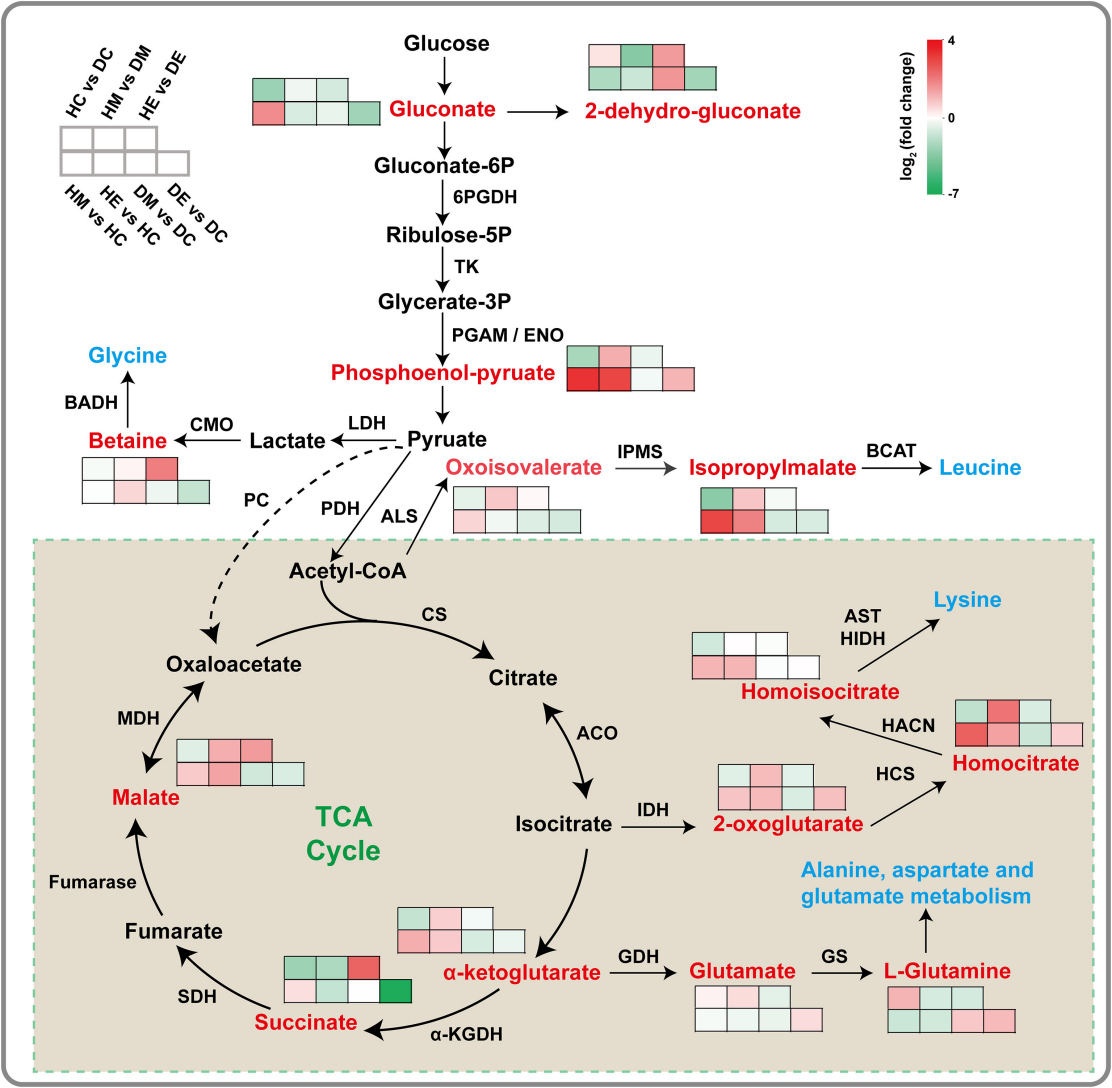
Comprehensive metabolic pathways of *L. chinensis* under saline-alkali stress. For the heatmap of each comparison group, the color gradient ranges from green to red, representing the Log_2_ (FC) values from low to high. HC, HM, and HE represent the relatively saline-alkali-tolerant cultivar ‘Huise’ (HS) under CK, MS, and ES treatments, respectively; DC, DM, and DE represent the relatively saline-alkali-sensitive cultivar ‘Dongbei’ (DB) under CK, MS, and ES treatments, respectively. For the comparison between different *L. chinensis* cultivars under the same saline-alkali stress, DB *L. chinensis* was used as the control; for the comparison of the same *L. chinensis* cultivar under different saline-alkali stress intensities, CK was used as the control.

There were both similarities and differences in the metabolic patterns of perennial *L. chinensis* plants with varying levels of saline-alkali tolerance, which might be associated with the self-regulatory mechanism through which *L. chinensis* mitigates saline-alkali stress (**Figure 11**). In the HC vs DC comparison, the abundance of L-glutamine was increased. In the HM vs DM comparison, the abundances of phosphoenol-pyruvate, oxidoisovalerate, isopropylmalate, 2-oxoadipate, and homocitrate were increased. In the HE vs DE comparison, the abundances of succinate and betaine were increased. The abundance of malate was increased in both the HM vs DM and the HE vs DE comparisons. The abundances of gluconate, oxoisovalerate, and succinate were increased only in the HM vs HC comparison. The abundances of phosphoenolpyruvate, homocitrate, and 2-oxoadipate were increased in the HM vs HC, HE vs HC, and DE vs HC comparisons. The abundance of betaine was increased only in the HE vs HC comparison. Isopropylmalate, homoisocitrate, α-ketoglutarate, and malate were significantly increased in abundance in the HM vs HC and HE vs HC comparisons but significantly decreased in abundance in the DM vs DC and DE vs DC comparisons. Notably, in the DM vs DC and DE vs DC comparisons, the abundance of L-glutamine was increased.

## 4 Discussion

### 4.1 Effects of saline-alkali stress on the growth and photosynthetic characteristics of *L. chinensis*

Morphological indicators, including plant height, root length, and biomass, can directly reflect plant growth status and productivity, and thus serve as key criteria for evaluating plant saline-alkali tolerance (Deinlein et al., 2014). In non-halophytes such as *Lolium perenne* (Cao et al., 2024) and *T. aestivum* (Guo et al., 2015), the synthesis of organic solutes such as Pro and SS represents a critical adaptive strategy for coping with saline-alkali stress. This process consumes substantial carbon sources and ATP, thereby reducing biomass accumulation (Liu et al., 2015). In this study, saline-alkali stress exerted similar inhibitory effects on root length, plant height, and leaf number of *L. chinensis*, but induced cultivar-specific differences in aboveground biomass. The aboveground biomass of DB *L. chinensis* showed a dose-dependent response to saline-alkali stress, while that of HS *L. chinensis* decreased only under the ES treatment. Furthermore, saline-alkali stress exhibited a ‘low-promotion and high-inhibition’ effect on chlorophyll synthesis; high-concentration stress inhibits the activities of key enzymes involved in chlorophyll synthesis, thereby lowering the photosynthetic rate and photosynthetic pigment content (Lin et al., 2017). Moreover, the decline in photosynthetic rate is regulated by stomatal or non-stomatal limitations (Kalaji et al., 2016). Stomatal limitation is characterized by a simultaneous decrease in *Gs* and *Ci*, whereas non-stomatal limitation shows reduced *Gs* but increased *Ci* (Flexas et al., 2004). In addition, Car functions dually as a photosynthetic pigment and a non-enzymatic antioxidant; specifically, Car can quench reactive oxygen species (ROS) and alleviate membrane lipid peroxidation (Fan et al., 2012). In this study, *Gs*, *Ci* and Car contents in DB *L. chinensis* displayed dose-dependent responses to saline-alkali stress; by contrast, the corresponding parameters in HS *L. chinensis* showed no significant differences between the MS and ES treatments. This indicates that HS *L. chinensis* possesses stronger antioxidant capacity. It is tentatively proposed that the decline in photosynthetic rate of DB *L. chinensis* is mainly caused by stomatal limitation, and this speculation requires further validation.

### 4.2 Effects of saline-alkali stress on the physiological characteristics of *L. chinensis*

Saline-alkali stress induces massive ROS accumulation, exacerbates membrane lipid peroxidation, and consequently impairs the structural stability of cell membranes. The contents of MDA and H_2_O_2_ are critical indicators for evaluating the degree of membrane lipid damage, and their levels rise with increasing saline-alkali stress intensity (Dinneny, 2015). In this study, the increased proportion of MDA content in DB *L. chinensis* (55.37%) was higher than that in HS *L. chinensis* (37.75%), suggesting that DB *L. chinensis* suffered more severe membrane lipid injury. Salt stress mainly disrupts plant metabolism via excessive ion accumulation, whereas alkali stress primarily disturbs rhizosphere environmental homeostasis and further impairs root function. Therefore, chloride-sulfate saline-alkali soil was used for stress treatment in this study. With the increase in saline-alkali stress intensity, soil pH and the proportion of alkaline ions increased synchronously, and their synergistic effect further aggravated growth inhibition and metabolic disturbance in *L. chinensis*. Ion toxicity triggers excessive intracellular Na^+^ accumulation and K^+^ depletion, thereby causing osmotic imbalance in plants (Hasanuzzaman et al., 2018). Moreover, Na^+^ accumulation in roots can be transported to leaves via transpiration, further elevating the leaf Na^+^/K^+^ ratio. The relatively saline-alkali-tolerant HS *L. chinensis* exhibited significantly lower Na^+^ accumulation in leaves and roots than the saline-alkali-sensitive DB *L. chinensis*. Combined with the metabolic differences in root exudates, *L. chinensis* cultivars with contrasting saline-alkali tolerance can indirectly regulate root ion absorption by modifying the metabolic composition of root exudates (Lin et al., 2022). Accordingly, the inter-cultivar difference in Na^+^ accumulation may result from the coordination of intrinsic tolerance mechanisms and root-level salt exclusion. Future studies could further clarify the synergistic contribution of these two adaptive strategies by integrating root ion transport and rhizosphere ion dynamics. Furthermore, Na^+^ contents in roots and leaves of *L. chinensis* continuously increased with rising saline-alkali stress intensity, while the K^+^ content showed a declining trend. The magnitude of ion increase in leaves was lower than that in roots, indicating that roots were more sensitive to ion perturbation. Continuous Na^+^ accumulation in root cells caused severe ion toxicity, which eventually reduced root vitality in *L. chinensis* (Fang et al., 2021). Plants maintain intracellular ROS homeostasis by activating antioxidant defense systems to protect cells from oxidative damage (Mir et al., 2018). SOD scavenges excess ROS to produce H_2_O_2_, while CAT and POD further decompose H_2_O_2_ into H_2_O and O_2_ (Willekens et al., 1997). This ROS-scavenging cascade alleviates excessive ROS accumulation rather than inhibiting ROS production, thereby mitigating ROS-mediated cell injury (Van Zelm et al., 2020). Under moderate saline-alkali stress, the activities of CAT, POD, and SOD remained at relatively low levels in *S. bicolor* seedlings (Sun et al., 2019) and *Zea mays* (Fu et al., 2017); with increasing stress intensity, these enzyme activities increased correspondingly. In this study, the activities of POD, CAT, and SOD in *L. chinensis* increased synchronously with rising saline-alkali stress intensity, which alleviated oxidative damage induced by saline-alkali stress. These results were consistent with previous reports. Saline-alkali stress damages cell membrane structure, increases membrane permeability, and disrupts plant osmotic balance. Plants sustain osmotic homeostasis by regulating the accumulation of osmotic regulators (Guo et al., 2019). Proline acts as a crucial osmolyte for maintaining cellular osmotic pressure and generally accumulates under abiotic stress (Hayat et al., 2012). The accumulation of low-molecular-weight osmolytes, including Pro, SS, and SP, constitutes an effective adaptive strategy to alleviate environmental stress damage (Farooq et al., 2009). Under saline-alkali stress, non-halophytes such as *S. bicolor* (Sun et al., 2019) and *T. aestivum* (Guo et al., 2015) accumulate higher levels of Pro and SS to relieve osmotic stress. In this study, the content of Pro, SS, and SP in *L. chinensis* increased markedly under saline-alkali stress, with a greater increase magnitude in HS than in DB. These findings suggest that HS *L. chinensis* possesses stronger osmotic regulation capacity. Previous studies have also demonstrated that the contents of SS, SP, and Pro in *L. chinensis* rise with increasing saline-alkali stress intensity (Evelin et al., 2019), which is consistent with our present results.

### 4.3 Effects of saline-alkali stress on the root exudates of *L. chinensis*

Under saline-alkali stress, plant roots secrete a variety of bioactive compounds; primary and secondary metabolites are potentially linked to variations in plant stress performance (Canarini et al., 2019). Moreover, plant cultivars with distinct saline-alkali tolerance differ considerably in root exudate composition (Hong et al., 2022), as well as in root biomass and root morphological traits under identical stress conditions (Cao et al., 2024). In this study, saline-alkali stress induced obvious metabolic remodeling in *L. chinensis* at the regreening stage. A wide range of metabolites were identified in root exudates, mainly including organic acids, amino acids, and lipids. Significant differences in root exudate profiles were observed between the two *L. chinensis* cultivars, and the number of DAMs was relatively lower in DB than in HS. This discrepancy may be partly attributed to the aqueous solution extraction method adopted for root exudate collection. Although this method is suitable for comparative analysis of metabolic components among cultivars, it separates root systems from natural rhizosphere soil and microbial interactions. This approach easily causes the loss of certain exudate components, induces unnatural secretion patterns, and may also introduce contamination from intracellular root metabolites, making it difficult to fully reflect the real root secretion characteristics under field conditions (Williams et al., 2021). Based on DAM screening, 13 core differential marker metabolites were further identified by integrating VIP value, fold change (FC) value, and intergroup significance. These metabolites exhibited stable expression patterns and clear intergroup differentiation across different cultivars and saline-alkali treatments, and can be regarded as key indicator metabolites. They provide a core theoretical basis for revealing metabolic differentiation among *L. chinensis* cultivars under saline-alkali stress (Yan et al., 2023). Accumulating evidence has confirmed that analyzing DAM markers and metabolic pathways can effectively reflect plant physiological status during growth and stress acclimation (Lin et al., 2022). Organic acids can reduce rhizosphere soil pH; for example, acetic acid is correlated with improved mineral availability via abiotic dissolution (Keiluweit et al., 2015), while amino acids are associated with cell membrane protection against oxidative stress and protein structure stabilization (Li et al., 2019). In this study, L-dihydroanticapsin (Organic acid) a DAM marker in the DM vs DC comparison, is a characteristic metabolite produced by endophytic *Bacillus velezensis*. This compound possesses antimicrobial activity (Tsalgatidou et al., 2022), and previous studies noted its potential correlation with plant growth and abiotic stress performance (Nifakos et al., 2021). In addition, carbohydrates, aromatic compounds, and tannin-related metabolites are significantly correlated with plant stress (Zhang et al., 2021). [8]-Shogaol (Benzene) a DAM marker in the HE vs HC comparison, can inhibit ROS production and scavenge superoxide anions and hydroxyl radicals (Dugasani et al., 2010); however, few studies have focused on its physiological function in plant stress responses. Scyllo-inositol (Alcohol) is a DAM marker in the HM vs DM comparison. In medical research, scyllo-inositol inhibits human telomerase activity and is considered a potential therapeutic candidate for Alzheimer’s disease (Ramp et al., 2021). Furthermore, scyllo-inositol hexakisphosphate is an important component of soil organic phosphorus and shows promising ecological potential for soil remediation (Turner et al., 2005). Pikuroside (Tannin) is a DAM marker in the HE vs DE comparison. Tannins can alleviate neuroinflammation and improve mitochondrial function (Zhao et al., 2025), selectively inhibit pathogenic bacteria, and promote the proliferation of beneficial microorganisms involved in nitrogen fixation and organic matter decomposition (Lang et al., 2025). Nevertheless, the physiological roles of pikuroside in plants remain poorly understood. Collectively, these DAM markers are correlated with stress-related metabolic shifts in *L. chinensis* under saline-alkali stress.

**Figure 12:**
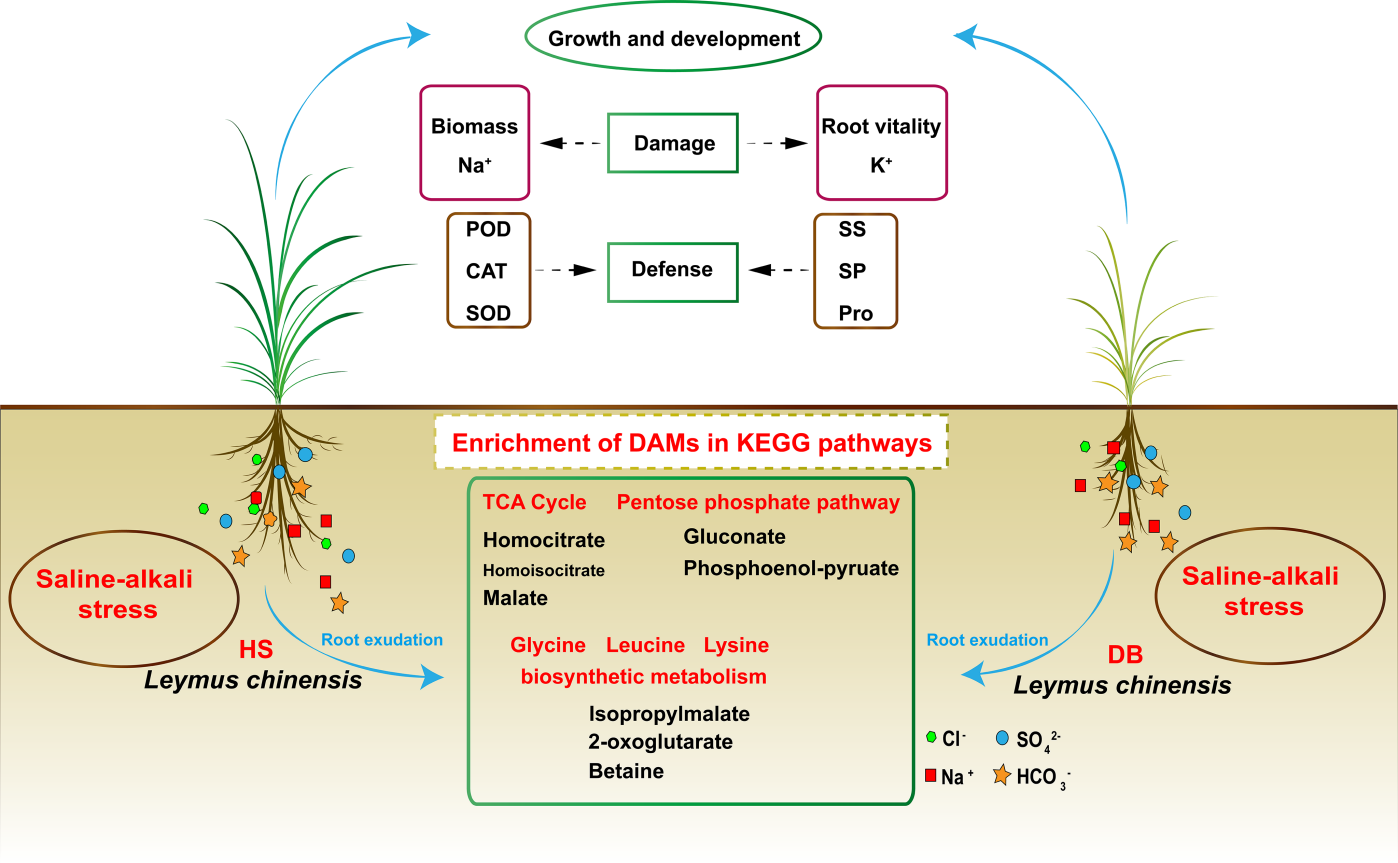
Metabolic responses of different *L. chinensis* cultivars to saline-alkali stress.

Pyruvate metabolism was significantly enriched in multiple comparison groups. As a pivotal hub connecting carbohydrate, lipid, and protein metabolism, this pathway modulates metabolic profiles under saline-alkali conditions, which is associated with the maintenance of cellular homeostasis in *L. chinensis* (Le et al., 2021). In this study, metabolites including gluconic acid, phosphoenolpyruvate, malic acid, succinic acid, citric acid, and isocitric acid were significantly enriched in energy metabolism pathways, which mainly participate in the pentose phosphate pathway (PPP) and tricarboxylic acid (TCA) cycle. The PPP provides essential precursors for nucleotide biosynthesis and generates reducing power. Its intermediates can be converted into glycolytic intermediates to produce ATP, thereby supplying material and energy support for plant growth (De Tredern et al., 2021). Furthermore, NADPH produced by the PPP is indispensable for photosynthetic electron transport and contributes to oxidative stress scavenging (Bolanos and Almeida, 2010). The TCA cycle is the core pathway of plant aerobic respiration, providing carbon skeletons and energy for multiple biosynthetic processes (Niehaus, 2021). KEGG enrichment analysis revealed obvious metabolic enrichment of the PPP and TCA cycle under saline-alkali conditions, indicating that these two pathways undergo remarkable metabolic reprogramming during the saline-alkali response of *L. chinensis*. Further pathway analysis showed that isopropylmalate (leucine biosynthesis) and betaine (glycine biosynthesis) were upregulated in HS but downregulated in DB. This finding indicates distinct differences in the biosynthesis of leucine and glycine between the two *L. chinensis* cultivars. Previous studies have shown that such amino acids are released into the rhizosphere via root exudation, and their presence is associated with buffered rhizosphere pH and modified alkaline soil properties. Meanwhile, as organic nitrogen sources and signal molecules, they optimize the rhizosphere microecology and alleviate alkali and ion toxicity (Li et al., 2019; Shafi and Shahid, 2025). Based on these findings, we speculate that compared with DB, HS establishes a more stable rhizosphere pH buffering system by accumulating and secreting free amino acids. By contrast, 2-oxoglutarate increased in both cultivars, while L-glutamine decreased only in DB. Insufficient glycolytic energy supply to the TCA cycle may correlate with constrained cellular energy pool, lowered glutathione abundance and intensified oxidative stress. In summary, we speculate that *L. chinensis* cultivars with different saline-alkali tolerance exhibit inter-cultivar differences at the metabolic pathway level under saline-alkali conditions. On the basis of the PPP and TCA cycle, HS *L. chinensis* presents differential metabolic changes in the leucine and glycine biosynthesis pathways, while DB *L. chinensis* mainly relies on the PPP and TCA cycle (**Figure 12**). These metabolic pathways have been proven to be enriched in *L. chinensis* under abiotic stress. The PPP, TCA cycle, and amino acid metabolism are stimulated to facilitate the synthesis of amino acids, secondary metabolites and carbohydrates, thereby helping plants cope with adverse environments (Lin et al., 2022; Wang et al., 2024a). At the transcriptional level, the upregulation of *dehydrin* and *LEA* genes, coupled with carbohydrate accumulation, can enhance the tolerance of *L. chinensis* to Na^+^ (Wang et al., 2020). Stress-induced genes are significantly enriched in multiple pathways including phenylpropanoid biosynthesis, amino acid metabolism, flavonoid biosynthesis and amino acid-related enzyme metabolism. A large number of genes associated with transport, hormone synthesis and transcriptional regulation have also been identified. Additionally, the expression levels of key genes involved in glycolysis, fatty acid synthesis and proteolysis are markedly increased (Wang et al., 2024b). This study fills the research gap regarding the differential responses of physiological, ionic, and metabolic pathways of root exudates in *L. chinensis* at the regreening stage under saline-alkali stress. In future research, we will combine 16S rRNA sequencing to verify the role of root exudates in recruiting beneficial microorganisms. Meanwhile, we will also conduct molecular functional verification of key metabolites and metabolic pathways, and carry out experiments in field saline-alkali environments to verify the applicability of the research results, thereby further improving the scientificity and practical value of this study.

## 5 Conclusion

Saline-alkali stress significantly inhibited the growth and photosynthetic physiology of *L. chinensis*, and there were significant differences in saline-alkali tolerance among different cultivars. Compared with DB *L. chinensis*, HS *L. chinensis* showed a smaller decrease in aboveground biomass. The pronounced reduction in photosynthetic rate of DB *L. chinensis* was primarily attributed to the dose-dependent changes of *Ci* and *Gs* along with increasing stress intensity. To alleviate the damage caused by saline-alkali stress, the antioxidant enzyme activities and the contents of osmotic regulatory substances in HS and DB *L. chinensis* increased simultaneously. Under saline-alkali stress, metabolic remodeling occurred in the PPP and TCA cycle of *L. chinensis*. DB *L. chinensis* mainly displayed metabolic changes in these two core energy metabolic pathways, while HS *L. chinensis*, in addition to these basic energy metabolism pathways, also exhibited obvious differences in the metabolic components related to amino acid synthesis. This study identifies inter-cultivar differences in the content, composition, and metabolic pathways of root exudates in *L. chinensis*, laying a theoretical foundation for breeding saline-alkali-tolerant cultivars.

## Data Availability

All the metabolomic raw data involved in this manuscript have been fully uploaded to the OMIX database of China National Center for Bioinformation (CNCB, official website: https://www.cncb.ac.cn/), with the accession ID PRJCA068007.
