# Supplementary figures and images for: Physiological characteristics and root exudate responses of Leymus chinensis to saline-alkali stress during the regreening stage
Source: Front Plant Sci. 2026 Jul 7;17:1844760. doi: 10.3389/fpls.2026.1844760 (PMC13389160; doi:10.3389/fpls.2026.1844760)

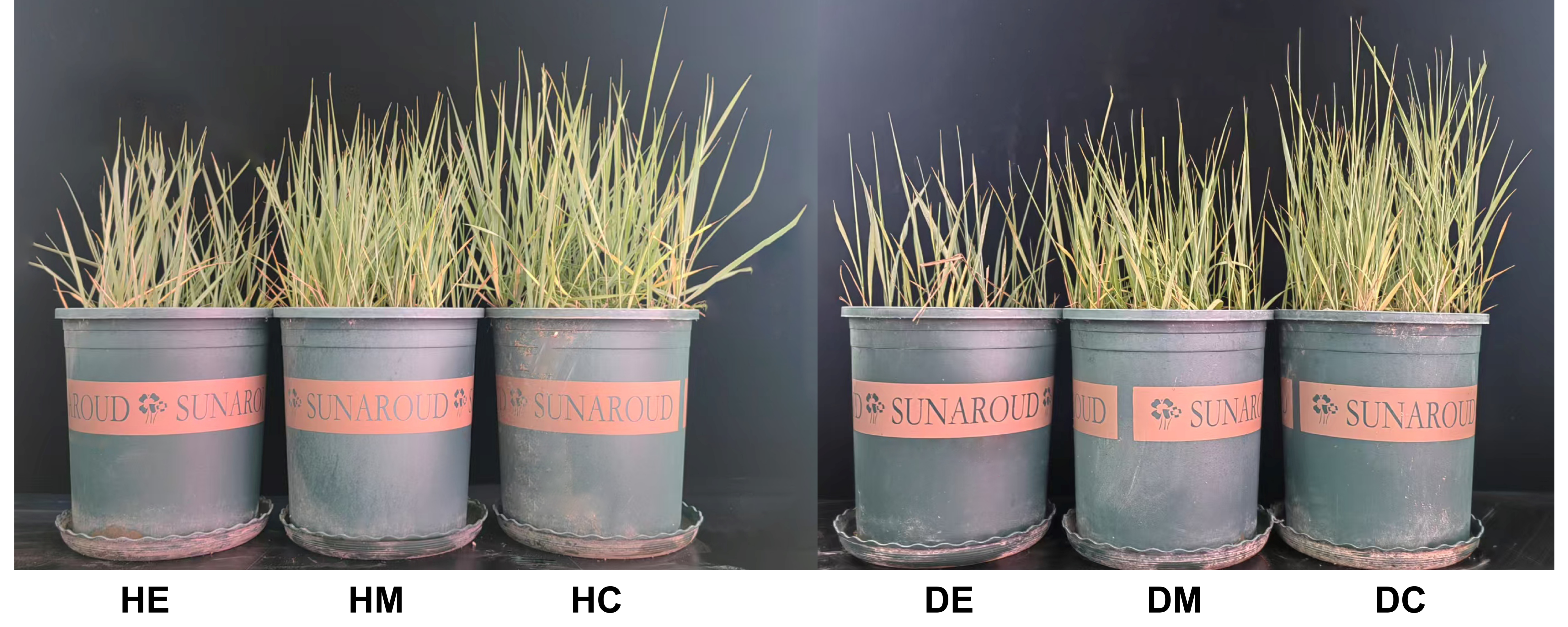

Supplement: Supplementary file 1 [file Image1.tiff]
